# Soil Lead Risks Associated With Urbanization Histories in Springfield MA and Hartford CT, USA

**DOI:** 10.1029/2026GH001854

**Published:** 2026-06-15

**Authors:** N. Perdrial, S. L. Walser, E. C. Sirkovich, J. B. Richardson, M. Cope

**Affiliations:** ^1^ University of Vermont Geography and Geosciences Burlington VT USA; ^2^ GUND Institute for the Environment Burlington VT USA; ^3^ Department of Geosciences University of Massachusetts Amherst Amherst MA USA; ^4^ Department of Environmental Sciences University of Virginia Charlottesville VA USA

**Keywords:** kriging, X‐Ray fluorescence, Redlining, environmental justice

## Abstract

Due to legacy leaded products, soil Pb is generally higher in older urban centers triggering substantial implications for environmental equity. By integrating a gridded soil Pb analysis of about 150 samples each in two historically industrial cities (Hartford, CT and Springfield, MA) with block‐level census data, we tested the hypotheses that (a) high soil Pb areas correlate spatially with older housing, (b) historical processes of discrimination have caused long‐lasting environmental injustices with respect to Pb exposure, and (c) multiple social, demographic and geographic factors intersect in determining areas in need of targeted remediation efforts. Our data and geospatial analysis showed higher Pb concentrations in Hartford than Springfield and confirmed the prevalence of elevated (>200 ppm) Pb in soils closer to older homes in both cities. Using a decision tree statistical partition, we show that exposure to elevated soil Pb was most prevalent in communities with children population higher than the city median, who live in multi‐family housing. In Springfield, ethnicity was a significant factor in exposure as census blocks populated by non‐Hispanic Whites were least likely to contain lead in soils above 200 ppm. Our analysis highlights that historical discriminatory practices, including redlining, have anchored environmental injustices in the studied communities, creating an invisible legacy challenge recorded in the land and carried across decades. The use of decision trees in the context of soil lead contamination provides a new method to help identify vulnerabilities of marginalized populations, providing quantitative tools to advocate for targeted mitigation and remediation.

## Plain Language Summary

Our analysis of soils in Hartford, CT and Springfield, MA reveals elevated soil lead levels that are associated with older houses. Using recent census data we show that Hispanic households and communities of color, particularly those living in multi‐family housing, are most at risk of soil lead exposure in these two cities. We argue that these vulnerabilities are remnants of past discriminatory practices in urban contexts, including redlining policies.

## Key Points

- Census block geospatial analysis allows for sub‐km^2^ statistical analysis of soil Pb risks by population characteristics
- Past discriminatory practices continue to exacerbate environmental injustices with respect to soil Pb in two New England cities
- Sub‐km^2^ geospatial analysis of soil Pb helps identify vulnerable communities

## Graphical abstract

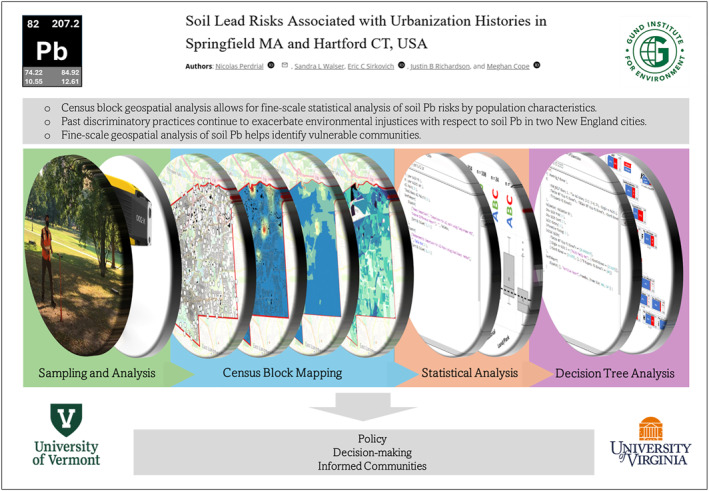

## 1 Introduction

The ubiquity of environmental soil contaminants necessitates precise determination of the factors influencing their distribution and effects on various communities. Spatial distribution of contaminants within urban settings can be difficult to delineate due to the heterogeneous nature of urban ecosystems, as well as the complex behavior of trace metals in soil (Reeder et al., 2006). Urban soils and their associated green spaces provide many ecosystem services, including decreased urban heat island effect, aesthetics, and trace metal sequestration (Cheng et al., 2015). While sequestration of trace metals by urban soils is beneficial for preventing mobilization as dust or in runoff, the high residence time of many trace metals in soils causes them to accumulate, thereby becoming a source of contamination to the public through ingestion and inhalation (Resongles et al., 2021). The industrial history and high urban density of the Northeastern USA released large quantities of trace elements in soils, including As, Cd, Cu, Hg and Pb. Among these, Pb in particular persists as a significant issue largely due to the combined widespread remains of legacy Pb‐containing paint and gasoline in the US (Bower et al., 2017; Clark et al., 2006; Clark & Knudsen, 2013; Henry et al., 2015; Mielke et al., 2005). Across the world, differing epidemiological and toxicological informed guidelines for Pb soil monitoring help identify contamination hotspots, target remediation efforts, and overall address Pb contamination. In the USA, the federal Environmental Protection Agency (EPA) lowered the soil screening level for Pb in residential areas from 400 to 200 mg/kg in 2025 (Busterud, 2025), based on a 5‐point composite sample (US EPA, 2007). However, some individual states have set their own levels, such as, for example, California (80 mg/kg—DTSC, 2020) or Vermont (41 mg/kg—VDH, 2023).

Chronic, asymptomatic low‐exposure to Pb is detrimental to the central nervous system, particularly in young children, while rare acute Pb toxicity can damage essential organs and systems such as the nervous, renal, and pulmonary systems (Wani et al., 2015). Although health agencies worldwide have sequentially lowered the acceptable blood Pb level since the 1960s (3.5 μg/dL for the US CDC, 5 μg/dL for WHO), they acknowledge that there is no safe level, with neurocognitive effects and other adverse health effects reported at low blood lead levels (BLLs) (ATSDR, 2020; EFSA, 2013; JECFA, 2011). To date, since the realization that Pb contamination greatly contributes to cognitive impairment, considerable improvements have been made in some parts of the world resulting in a measured decrease in Elevated Blood Lead Levels (EBLL) between generations (McFarland et al., 2022). However, Pb remains largely problematic worldwide with some estimating that EBLL contributes to almost 1% of the global burden of disease (Fewtrell et al., 2004).

Lead poisoning was recognized early by authors such as Vitruvius or Pliny the Elder (see references in Retief & Cilliers, 2005). In 1839, in the *Traité de Maladies de Plomb ou Saturnine*, Louis Tanquerel des Planches (see reference in Seaton, 2014) described many cases of lead poisoning due to occupational exposure. Over 130 years following this work, Herbert Needleman's work on the effects of high Pb in children's tissues (Needleman et al., 1979) led to the slow realization that environmental Pb was directly correlated to neurological deficit in children. Following work on soil Pb sources by Mielke et al. in Baltimore (Mielke et al., 1983) and Minneapolis (Mielke et al., 1984), research pinpointed a spatial control over high lead concentrations in soil, associated with high traffic and older areas in cities. Today, it is well‐accepted that lead‐paint, household dust, and past deposition of leaded gasoline are the predominant Pb sources in soils in urban systems (Henry et al., 2015; O'Shea et al., 2021; Wade et al., 2021). By 1994, results from the ban and rapid phase‐down of leaded gasoline initiated in 1988 in the US resulted in a significant decrease in BLL in children (Pirkle et al., 1994). Today, the combination of the Pb paint ban in 1977 (16 CFR § 1303) and the Pb gasoline full ban in 1996 (40 CFR § 80) has had beneficial effects on BLL in the US and the world (Cerling et al., 2026; Lacerda et al., 2023). Nonetheless, soil Pb remains generally higher in older urban centers (Filippelli et al., 2024; Frank et al., 2019; Mielke et al., 2005), which has significant implications for environmental equity. It has been shown that Pb poisoning has disproportionately affected Black and low‐income communities due to social, economic, and political practices that concentrate these groups in the oldest, least rehabilitated housing within a metro area (Needleman, 2004; Sargent et al., 1995; Yeter et al., 2020). This is further exacerbated by the fact that lower‐income communities are less able to afford Pb paint abatement for aging housing stock and—if they are renters—have little power to force landlords into compliance. A recent study estimated that, under the recent lowering of screening levels by the EPA, 30 million US households would need to mitigate potential soil Pb hazards, at a potential total cost of $290 billion to $1.2 trillion, further burdening low‐income communities (Filippelli et al., 2024).

The intertwined oppressions of racism and capitalism in the US—widely recognized as “racial capitalism”—actively concentrate communities of color and low‐income communities in less‐desirable sections of both urban and rural areas (Pulido, 2000, 2017). Racial capitalism is foundational to the iterative processes of environmental racism (Bullard, 1993), because it underlies the devaluation both of people and of places, resulting in a spiral of differential environmental quality and risks. As Bullard states: “Residential apartheid and skewed development policies limit mobility, reduce neighborhood options, diminish job opportunities, and decrease environmental choices” (Bullard, 1993, P24). Environmental racism therefore cumulatively favors white communities (especially middle‐class and wealthy ones), insulating them from negative views of their people and their places, in turn elevating their social, economic, and political standing. These interwoven benefits ensure white residents' access to green spaces, lead‐remediation dollars, and cleaner air, water, and soil. As shown in several recent studies, Pb is a particularly potent contaminant with respect to environmental justice (Dietrich, 2020; Ericson et al., 2021; Larsen & Sanchez‐Triana, 2023).

Predictive geospatial models, such as kriging or co‐kriging are used, within limits, to efficiently estimate soil Pb risks from limited sampling (Bower et al., 2017; Cattle et al., 2002; Goovaerts, 1999; Guinn et al., 2024; Solt et al., 2015; Triantafilis et al., 2001). The objective of this study was to use a novel decision‐tree model to evaluate the racial, socioeconomic, and place‐based factors influencing soil Pb exposure risks in two urban centers representative of the Northeastern US. We hypothesized that (a) historical processes of discrimination have caused long‐lasting environmental injustices with respect to Pb exposure, (b) multiple social, demographic and geographic factors intersect in determining areas in need of targeted remediation efforts, and that (c) a decision tree statistical model is a useful tool to decipher intersecting parameters in urban centers with complex histories. By testing these hypotheses and identifying the prevalence and factors governing soil Pb beyond simplistic geospatial models, our work can help design future predictive models needed for effective—and equitable—legislation and remediation.

## 2 Materials and Methods

### 2.1 Study Sites

**Figure 1:**
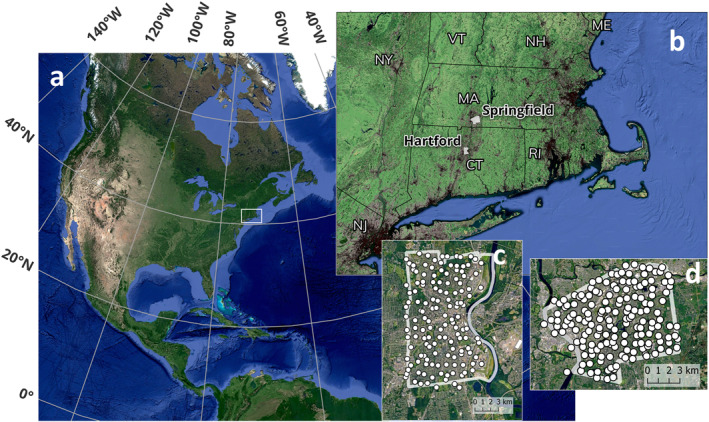
Location of the study areas within white rectangle in (a). Background on (b) is a simplified land cover map of forests and developed land. Satellite images from Google Satellite. Location of samples shown for Hartford (c) and Springfield (d) are from Sirkovich et al. (2023).

Two midsize cities in the Northeastern USA were selected for soil sampling: Hartford, CT (121,054 pop.), and Springfield, MA (155,929 pop., Table 1 and Figure 1). These were chosen due to their similar environmental variables (climate, geology), population size, housing characteristics, and comparable industrial histories. Despite being in different states, these cities are 25 miles apart and share an airport (BDL), and a connecting string of communities that act as an economic corridor and workforce conurbation. Both cities are between Dfa and Dfb Köppen climate classification, with humid continental mild to hot summers, no dry season, the coldest month averaging below −0°C, and sit on young soils developed on Pleistocene till. While Hartford and Springfield are similar with respect to population size, poverty rates, and income levels, they differ in their demographic makeup, with 79.5% of the population of Hartford identifying as Black and/or Hispanic, versus 67% of the population of Springfield (Table 1); their location in different states provides some comparative value to the extent that the state‐level policies of Massachusetts and Connecticut differ. The size, ethnic group proportion, and socioeconomic characteristics of the cities are provided on Table 1, and a detailed urbanization history of the two cities is provided in the Supporting Information S1. In short, both cities developed as industrial centers between the second part of the 19th century and the mid‐20th century. During this period they both acted as immigration centers in the region. Both cities experienced deindustrialization and economic restructuring after 1970, leading to modification of their demographics. Both cities experienced “white flight,” and both urban regions were subject to documented racist real estate (“redlining”) policies (Nelson et al., n.d.). Today, both cities have poverty rates twice the rate of their respective states (27.3% vs. 10.2% for Hartford, CT; and 25.0% vs. 9.7% for Springfield, MA—Table 1).

**Table 1:** City Size, Ethnic Group Proportion, and Socioeconomic Characteristics

City name	Hartford	Springfield
Population^a^	121,054	155,929
Population identifying as White, Not Hispanic/Latino^a^	12.62%	28.25%
Population identifying as Black or African American alone, Not Hispanic/Latino^a^	35.54%	20.84%
Population identifying as Hispanic/Latino (any race/races)^a^	44.0%	46.74%
Poverty rate^b^	27.30%	25.00%
Owner‐occupied rate^b^	23.50%	47.30%
Proportion of housing units built before 1960^b^	60.35%	65.05%
Yearly median income per capita^b^	$US 22,784	$US 23,161
Median value of owner‐occupied homes^b^	$US 173,200	$US 170,500
Median monthly gross rent^b^	$US 1,150	$US 964

^a^ Data from the US Census bureau (US Census Bureau, 2020a).

^b^ Data from the 2021 5‐year American Community Survey estimates (US Census Bureau, 2023).

### 2.2 Soil Sampling

The samples were previously collected and analyzed for trace element distribution (Sirkovich et al., 2023). We used ArcGis “Create 19 Fishnet” tool (ESRI, 2019) to generate an evenly spaced grid of approximately 150 sample areas per city. Within each area we identified an urban forest site for sampling. We define an urban forest as a site under tree cover which receives leaf litter regularly. We selected urban forests because the presence of trees indicates that these soils have been in place for a significant amount of time. Because they receive leaf litter regularly they also receive comparable inputs. In Spring 2021, we conducted visual inspections at each individual sampling site to ensure soils were at least 1 m away from pavement to avoid collection of soils biased by the presence of displaced materials. These inspections also prevented the collection of wet soils, soils with thick organic horizons, and soils with artificial influences. Once a sample site was identified, its coordinates were recorded, the O‐horizon material was scraped away, and at least 0.5 kg of soil from the surface to a depth of 10 cm was collected using an auger and stored in a polypropylene bag. A total of 140 and 168 samples were collected from Hartford and Springfield, respectively.

### 2.3 Soil Analysis

All soil analyses were performed ex situ, on air‐dried soils sieved to 2 mm. We used a SciAps X200 portable X‐ray Fluorescence (pXRF) analyzer, calibrated using standard reference materials: BCR‐2, BHVO‐2, DNC‐1, GSP‐2, SBC‐1, SDO‐1, STM‐2, W‐2, and W2‐A from USGS, and 1633b, 2709a and 2710a from the National Institute of Standards and Technology, well‐characterized soil samples and total digestions from the urban soils as described in detail in Sirkovich et al. (2023). A custom empirical calibration was set to 60s analysis time. Samples were measured four times each through zip‐lock polypropylene bags. To account for the heterogeneity of these soil samples, the position of the sample was adjusted between each measurement, and the results represent an average of quadruplicates, corrected for instrument variable on dry soils using the equation in Table S3 of Walser et al. (2023). The method of calibration and potential effects is described in Walser et al. (2023) and Sirkovich et al. (2023).

### 2.4 Geospatial Analysis

Geospatial analysis was completed with QGIS 3.28 (QGIS.org., 2026). To best integrate all aspects of the geospatial assessment, we chose to analyze data at the census block level which is the finest resolution of the US census and corresponds to areas bounded by visible features (street, roads, streams). Because geospatial data varied from point analysis (soil Pb values), parcels (housing age, valuation and land‐use) to census blocks (demographics), we extrapolated the data from finer granulometries (points and parcels) to the census block level.

Census blocks for the year 2020 for the two cities were downloaded from the U.S. Census Bureau TIGER database (US Census, 2020b) and joined in MS Excel to the census block demographic data for 2020 from the U.S. Census Bureau database (US Census, 2020a). For each census block, the non‐Hispanic White Alone population, non‐Hispanic Black or African American Alone population and Hispanic and Latino—Any Race population percentages were calculated, creating three discrete groups from here on referred to as: “non‐Hispanic White,” “non‐Hispanic Black,” and “Hispanic/Latino”). In addition, while the number of children under 18 years old was extracted from the “P3” data, the number under 5 years old was determined by summing each age class from the results of the 2021 5‐year American Community Survey estimates, which are only available at the coarser census “block group” level (ACS, 2021). Because the databases for children's ages are not available at the smaller census block level, some differences exist in the block coverage between these data sets.

As a proxy for Pb source, the distances between each sample and the nearest building and nearest road were determined using the “distance to nearest hub” algorithm in QGIS 3.28.9. For this, layers of roads and buildings were downloaded from the Connecticut DEEP GIS Open Data Website (CT DEEP, 2025) for Hartford and the UMass Design Center for Springfield (CAFE, 2025). Because the buildings and road layers were restricted to city boundaries, a few sample points lying outside of the city boundary were removed from the distance analysis results in both cities: this resulted in 18 Hartford samples (13% of the samples) and 12 Springfield samples (18% of the samples) being removed.

Discrete soil Pb analyses were subjected to ordinary kriging modeling using the SAGA 7.8.2. Ordinary kriging algorithm (Conrad et al., 2015). For both cities the raster interpolation cell size was set to 100 m × 100 m and the variogram was determined using a variance approach and a maximum search distance of 400 m. The resulting raster map was then subjected to the QGIS zonal statistics algorithm to extract the arithmetic mean Pb concentration within each census block.

Geolocated parcel‐level data for year of construction, tax valuation, and land‐use classification were acquired from the yearly assessor's database of each of the cities for fiscal year 2022 (MassGIS, 2026; Open Data Hartford, 2026). The median year of construction, median site valuation of parcels, and most common land‐use code within a census block were extracted using the “Join attributes by location (summary)” QGIS algorithm from the parcel layers to be joined with the census block layers. Because land use codes are complex and often state‐ or city‐specific, we simplified the codes into six main categories: (a) single family residential, (b) multi‐family residential, (c) commercial, (d) industrial, (e) land and parks (not including vacant lots) and (f) all other or unknown land‐uses.

The geospatial data set used in this study therefore consisted of census level polygonized data for: (a) averaged Pb soil values from interpolation in ppm (i.e., mg/kg), (b) population characteristics, (c) median housing age, (d) median valuation, and (e) most common land use codes.

### 2.5 Statistical Analysis

Statistical analysis of the data was focused on the pair‐wise comparison of means of estimated soil Pb concentrations within and between parameters. Three building age classes were chosen to be representative of Pb‐paint usage in the area, with a class of blocks dominated by buildings constructed prior to 1950, a class for blocks mainly built between 1950 and 1978 and a class representing blocks developed after the Pb‐paint ban of 1978. The intermediate class (1950–1978) was chosen to reflect the pre‐regulation phasing‐out of Pb‐paint products initiated by health professionals and consumer advocate groups in the USA in the late 1940s and early 1950s (Filippelli et al., 2015). Each demographic parameter was transformed into percent population in the census block and classified in 20% bins. We selected 20% bins as this led to the most evenly divided binning for all categories across the two cities. Tax valuation medians in census blocks were classified in four quartile classes based on their distribution in each city. Each quartile is set to represent exactly a quarter of the blocks, ranging from the lowest rate (first quartile) to the highest rate (fourth quartile). This allows for normalized observations irrespective of local tax estimates and markets.

For each data set, statistical significance of mean, excluding unknown values, was assessed using a Steel‐Dwass’ non‐parametric multiple pair‐wise comparison analysis computed on non‐log transformed data using JMP Pro 17.0.0 (JMP Statistical Discovery LLC, 2022). In all cases the significance level (*α*) was set at 5%. To represent significance between classes, in the main text of the manuscript, we report differences using connecting letters for simplicity where levels not connected by the same letter are significantly different. We chose the non‐parametric Steel‐Dwass' method (Hollander et al., 2015), instead of the more classic Tukey‐Kramer comparison because variance inflation factors revealed some collinearity in the variables (Table S1 in Supporting Information S1) and because some of the data is non‐normal. The procedure and full results of the statistical analyses, including *p*‐values are available in the Figure S4 of Supporting Information S1 (p. 8).

For the residential blocks only, we performed a decision tree (a.k.a. partition) analysis on categorical data using the decision tree module JMP Pro 17.0.0. We set the response variable as Pb in soils above or equal to 200 ppm and all predictors above or below their median value. The model was developed using 75% of the data as training and 25% as validation. We split the tree to the 5% significance level. For each leaf we determine the odds of the block to have soils above 200 ppm Pb by dividing the percent likelihood of being above 200 ppm for each leaf by the likelihood at the city scale. Details of the goodness of fit, including miscalculation rates, are available in the Figure S5 of Supporting Information S1 (p. 9).

## 3 Results

### 3.1 Concentration and Distribution of Pb in Urban Forests' Soils

**Figure 2:**
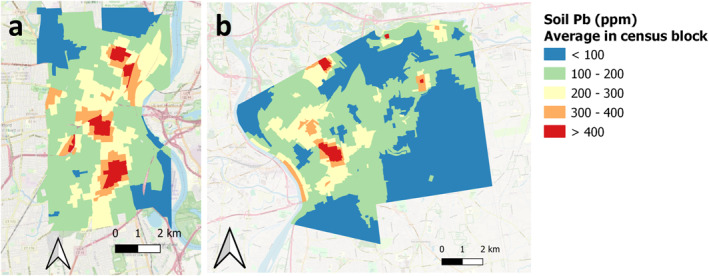
Distribution of Pb in census block soils extrapolated from individual samples published in Sirkovich et al. (2023) using ordinary kriging in panel (a) Hartford, CT and (b) Springfield, MA.

In Hartford (*n* = 140), Pb concentrations vary between 28 ± 3 ppm and 976 ± 61 ppm, corresponding to a mean of 193 ppm and a median of 142 ppm. Forty samples contained over 200 ppm Pb, the EPA screening value for residential soils (Figure 2). The soils with the highest Pb values were not concentrated in a single zone but rather in three separate areas (Figure 2a).

In Springfield (*n* = 168), Pb concentrations vary between 25 ± 10 ppm and 789 ± 75 ppm, corresponding to a mean of 131 ppm and a median of 93 ppm. Twenty‐seven samples contained over 200 ppm, principally in the downtown area and in three isolated locations (Figure 2b).

Guidelines for Pb in soils are highly variable between countries and within countries. While there are no state‐specific screening levels for Pb in soils in Connecticut, the State of Massachusetts Department of Environmental Protection published soil provision guidance stating that the maximum Pb concentration in soil for re‐use in residential areas, playgrounds, schools, or groundwater resource areas is 200 ppm (MassDEP, 2014), similar to the recently lowered EPA guidance (Busterud, 2025; Filippelli et al., 2024). Under this guidance, 17.5% of samples collected in Springfield are considered contaminated and 32.4% of the samples in Hartford are considered contaminated.

### 3.2 Census Block Analysis

#### 3.2.1 Building Age

**Figure 3:**
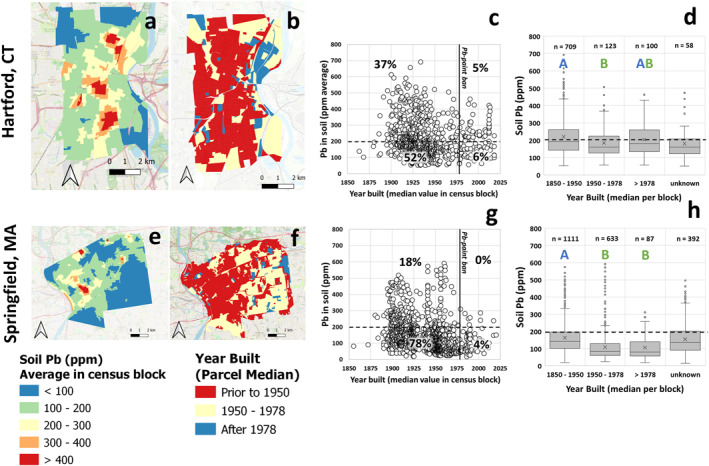
Extrapolated soil Pb (a, e), median age of buildings (b, f), soil Pb value relation to median year built (c, g) and box plots distribution of soil Pb values per median year of built (d, h). The resolution for all data is the census block. Dotted lines in panels (c, d, g, and h) correspond to the EPA screening level for Pb in soils (200 ppm). Percentages in (c) and (g) correspond to the percent of blocks belonging to the criteria bounded by the Pb screening level and the Pb‐paint ban (vertical line). Box plots in (d) and (h) represent the spread of data with the box containing the second and third quartiles, the cross the mean value, and the horizontal line within the box the median value. Only points outside of 1.5 times the intra‐quantile range are displayed for ease of reading. Connecting letters in (d) and (h) highlight significant differences where levels not connected by the same letter are significantly different as determined by the Steel‐Dwass non‐parametric multiple pair‐wise comparison analysis (*p* < 0.0001).

Integration of the soil Pb data at the census block level (Figures 3a and 3e) allows us to assess the correlation between Pb concentration and building age. While the age of structures varies within each city, the majority of construction dates occurred prior to the Pb paint ban of 1978 (Figure S2 in Supporting Information S1). In both cities, the highest estimated soil Pb corresponds to blocks dominated by pre‐1978 housing (Figures 3c and 3g). Only 5% and 0.4% of the post‐1978 blocks are expected to contain over 200 ppm Pb in the soil in Hartford and Springfield, respectively (Figures 3c and 3g). Thirty‐seven percent of all census blocks in Hartford and 18% in Springfield are dominated by pre‐1978 buildings and contain over 200 ppm soil Pb (Figures 3c and 3g). Box‐and‐whiskers plots reveal generally low mean Pb concentrations in more recently built blocks (Figures 3d and 3h), as well as large numbers of high values (outside of the box) in older blocks that are representative of the hot‐spot distribution shown on the maps (Figures 3a and 3e). Omitting the unknown aged blocks (6% in Hartford, 18% in Springfield, Figure S2 in Supporting Information S1), the average and median soil Pb values typically trend downwards with decreasing housing age, with a significant difference between the mean of blocks dominated by pre‐1950 structures compared to 1950–1978 structures (Figures 3d and 3h). In the case of Hartford, the newer construction blocks' mean Pb is not statistically significantly different than either of the other groups' (Figure 3d). In Springfield, the newer construction blocks' Pb levels are similar to the 1950–1978 blocks and significantly lower than the Pb concentration in soil of the older blocks (Figure 3h).

#### 3.2.2 Demographics

The demographic makeup of Hartford and Springfield shows blocks dominated by non‐Hispanic White populations (west blocks in Hartford, South and East Springfield, Figures S3c and S3d in Supporting Information S1) and others by non‐Hispanic Black populations (North Hartford, Springfield Old and Upper Hills, McKnight, Figures S3e and S3f in Supporting Information S1) and Hispanic/Latino populations (South Hartford, Springfield's River banks and downtown, Figures S3g and S3h in Supporting Information S1).

**Figure 4:**
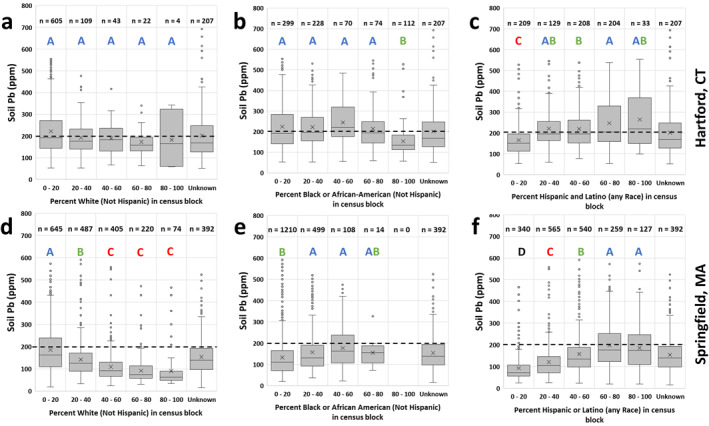
Correspondence between extrapolated soil Pb and population demographics in census blocks for Hartford (a–c) and Springfield (d–f). Dashed lines in the box plots correspond to the EPA screening level for Pb in soil (200 ppm). Box plots characteristics are the same as in Figure 3. Letters in the plots represent significance where levels not connected by the same letter are significantly different (*p* < 0.001).

In Hartford, there is no significant difference in the soil estimated mean Pb value as a function of the non‐Hispanic White population distribution (Figure 4a). However, in Springfield, the mean soil Pb is significantly lower in blocks with 20%–40% non‐Hispanic White residents and with over 40% non‐Hispanic White residents (Figure 4d). In Hartford, blocks with 0%–60% non‐Hispanic Black residents have a similar mean soil Pb concentration at around 200 ppm. However, blocks with 80%–100% of that population have a significantly lower mean soil Pb concentration (Figure 4b). In Springfield, blocks with the lowest (0%–20%) proportion of non‐Hispanic Black residents have significantly less soil Pb than blocks with 20%–60% of that population (Figure 4e). There are not enough blocks with over 60% non‐Hispanic Black residents in Springfield to be statistically meaningful. The clearest relationship between demographics and mean soil Pb in both cities concerns the Hispanic/Latino (any Race) residents. In both cities, blocks with the lowest Hispanic/Latino population (0%–20%) have the lowest mean soil Pb (151 and 72 ppm in Hartford and Springfield, respectively, Figures 4c and 4f). In both cities the estimated mean soil Pb value increases steadily and significantly with increasing Hispanic/Latino population to reach 264 ppm on average in blocks with 80%–100% of these residents in Hartford (Figure 4c) and 185–198 ppm on average in blocks with 60%–100% of these residents in Springfield (Figure 4f).

#### 3.2.3 Child Population

Because the census block group (within the American Community Survey) is the smallest geographic unit at which children are counted by age, there is no data at the census block level. To compare with other demographic data, we therefore geographically attributed the block group values to the blocks they contain (Figure S4 in Supporting Information S1). While this partially departs from the results presented above, it is relevant to analyze the relationship between estimated soil Pb and the reported number of children of young age within census blocks as children are the most vulnerable population (Wani et al., 2015). Here, we chose to explore the relationship between estimated soil Pb and the number of children <5 years old (yo) and the number of youth (<18 yo) in census blocks. In both cities, census blocks with a low percentage of residents aged less than 5 yo, have significantly lower Pb concentrations than blocks with higher percentages (Figures S3e and S3f in Supporting Information S1). In Hartford the difference is statistically significant between blocks with less than 10% residents under 5 yo (Pb mean: 198–213 ppm) and blocks with over 10% residents under 5 yo (mean Pb: 241–289 ppm). In Springfield the same trend is observed at a higher population cut‐off of 15% residents under 5 yo with a Pb mean of 137–146 ppm for blocks with less than 15% residents under 5 yo and a Pb mean of 183 ppm for blocks with over 15% of the population under 5 yo. When considering the proportion of all young residents in the blocks, estimated soil Pb clearly increases with higher proportions of children in both cities. In Hartford, there is significant Pb mean difference between blocks with less than 30% of the residents under 18 yo (186–203 ppm) and blocks with 30%–40% of the residents under 18 yo (259 ppm) and over 40% of the population under 18 yo (329 ppm). In Springfield, the mean Pb value is significantly lower in blocks with less than 10% residents under 18 yo at 102 ppm, compared to blocks with 10%–40% of their population under 18 yo at 143–150 ppm and blocks with over 40% of residents under 18 yo at 207 ppm.

#### 3.2.4 Socio‐Economic Factors and Land‐Use

**Figure 5:**
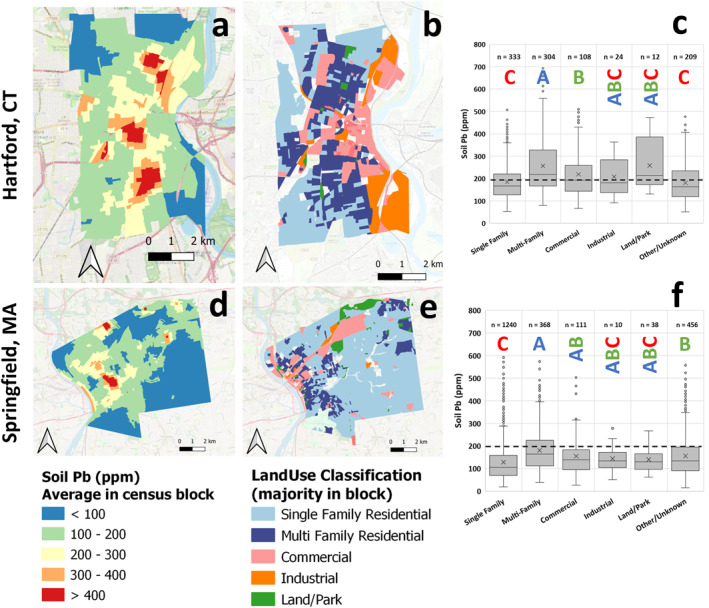
Correspondence between extrapolated soil Pb, simplified land‐use classification in census blocks and statistical significance in Hartford (a–c) and Springfield (d–f). Dashed lines in the box plots correspond to the EPA screening level for Pb in soils (200 ppm).

We used a simplified land‐use classification and city‐derived tax valuations to explore the connection between soil Pb and socio‐economic factors (Figure 5 and Figure S4 in Supporting Information S1). Our simplified land‐use classification differentiated residential, commercial, industrial, and natural areas. Within the residential denomination we discerned single‐family and multi‐family residential as a proxy for economic status as the majority of single‐family homes in the US are owner occupied while multi‐family residential buildings tend to be renter occupied (Pfeiffer et al., 2021). In both cities, commercial blocks are clustered in the historical downtown areas, lining the principal streets, or along river areas. Similarly, multi‐family housing tends to be clustered in downtown areas with isolated blocks in the periphery (Figures 5b and 5e). While the numbers of multi‐family dominated census blocks in Hartford and Springfield are comparable (approx. 300 census blocks), there are significantly more census blocks dominated by single family residential parcels in Springfield (1,240) than in Hartford (333). In both cities, the mean soil Pb values of commercial, industrial, and land/park dominated blocks are not significantly different from other blocks (Figures 5c and 5f). With respect to residential blocks, both cities show a significant difference in mean Pb values for blocks dominated by single family versus multi‐family dwellings (Figures 5c and 5f). In both cases the mean soil Pb value is higher in multi‐family blocks compared to single‐family blocks, with a difference between 186 and 257 ppm for Hartford and 128 ppm and 180 ppm for Springfield (Figures 5c and 5f).

Tax valuation is highly dependent on land‐use classification, as commercial and industrial properties are taxed higher than residential properties in Hartford and Springfield (Offices of the Assessor, Hartford, CT and Springfield, MA). For that reason, we performed the tax analysis only on blocks dominated by residential properties (Figure S5 in Supporting Information S1). While this provides some level of comparison, because single family and multi‐family are analyzed together, it does not necessarily truly reflect financial consideration of the occupants but rather of the owners. For both cities, there is a significant difference in the average soil Pb between blocks taxed at the various quartiles (Figure S5 in Supporting Information S1). The relationship is, however, inverted between the two cities as soil Pb estimates are higher for the third quartile blocks compared to the first and second quartile blocks in Hartford, while in Springfield the opposite is observed (Figure S5 in Supporting Information S1).

## 4 Discussion

### 4.1 Distribution and Sources of Pb

The distribution of soil Pb in both cities is characterized by the presence of hotspots in the oldest areas of the cities (Figures 2a and 2e). Although the maximum Pb concentration was higher in Hartford than Springfield, the range of values was similar (i.e., between 25 and 1000 ppm) in both cities. Such values for Pb in urban soils are typical of Northeastern US cities (Bower et al., 2017; Datko‐Williams et al., 2014; Laidlaw et al., 2017; Sirkovich et al., 2023), with the highest values typically present in historical areas, often located in downtowns.

In both cities, the distribution of Pb corresponded well with the population density which reflects the density of buildings and roads, two of the main sources of legacy Pb in soils from either Pb‐paint weathering or Pb‐gasoline aerosols from vehicles (Filippelli et al., 2015). While sourcing of Pb in soils can be achieved using Pb isotopes, primary sources can be estimated by analyzing the spatial correspondence between potential sources and Pb distribution (Wang et al., 2022). Indeed, because Pb is relatively immobile in the soil system, its transport remains limited to atmospheric dust in urban settings (Allison & Allison, 2005; Laidlaw & Filippelli, 2008). Analysis of the distance between the sampling points and the nearest building and road (Figure S6 in Supporting Information S1), show that points sampled far away from buildings and roads always have Pb concentrations below 200 ppm (except for one sample in Hartford containing 219 ppm Pb and located 136 m from a road and 54 m from a building). On the other hand, the highest soil Pb concentration in both cities corresponded to samples taken within 50 m of a building or 50 m of a road. The relationship between average building age and soil Pb at the census block level clearly indicated that soils from areas with pre‐1950 development are more likely to contain higher soil Pb concentrations (Figure 3). In Hartford, however, the blocks dominated by post‐1978 development were not statistically different from older blocks (Figure 3d). We attribute this to the fact that some of the newer blocks in Hartford are located in the historic river edge district close to Pb hot spots (Figures 3a and 3b). Whether the high Pb is due to building age or accumulation of Pb from road traffic over time cannot however be deciphered with this data set as both factors are linked and based on historical urban development patterns in both roads and buildings. In both cities, the source of Pb did not appear to come from industrial activities (Figure 5) but rather from legacy use of Pb‐paint and gasoline; because of the statistical difference in soil Pb and building age, we suspect legacy Pb from paint and gasoline is the primary source of Pb in both Hartford and Springfield. This prevalence of legacy Pb as a source is widespread in urban systems in the USA such as Durham, NC (Wang et al., 2022), Appleton, WI (Clark & Knudsen, 2013) and elsewhere (see Burgoon et al., 1995).

### 4.2 Environmental Justice Implications and Risk Disparity

#### 4.2.1 Increased Vulnerability for Hispanic/Latino Communities

Analysis of soil Pb mean concentrations as a function of demographic characteristics showed slightly different patterns in Hartford and Springfield. In both cities the mean soil Pb concentration was significantly higher where the proportions of Hispanic/Latino populations in the census block were higher (Figures 4c and 4f). A contrario, the mean soil Pb concentration is lower in areas with higher proportions of non‐Hispanic White residents in the census block (Figures 4a and 4d); however, this appeared to be only statistically significant in Springfield. This difference between the two cities is potentially due to the lower diversity in population in Springfield (28.25% residents identifying as non‐Hispanic White—Table 1) versus Hartford (12.6% residents identifying as non‐Hispanic White—Table 1). Indeed, maps of the non‐Hispanic White demographic distribution between the two cities (Figures S3c and S3d in Supporting Information S1) show that this population in Hartford is more widely distributed than in Springfield. Further, the clear correspondence in Springfield between the distribution of non‐Hispanic White residents (Figure S3d in Supporting Information S1) and the distribution of building age (Figure 3f) indicates that this population tends to concentrate in residential areas developed after the Pb‐paint ban, effectively lowering their vulnerability.

The difference in vulnerabilities to Pb for the non‐Hispanic Black population between the two cities (Figures 4, 5, and 5e) can be explained by the distribution and prevalence of that population. In Hartford, 36% of the population identifies as non‐Hispanic Black; this population is largely concentrated in the northern part of the city (Figure S3e in Supporting Information S1). Comparatively, in Springfield, 21% of the population identifies as non‐Hispanic Black and is distributed across the city with some concentration in the older part of the city (Figure S3f in Supporting Information S1). The clear geographic segregation of Hispanic/Latino and non‐Hispanic Black populations in Hartford seems to correlate with increased vulnerability of these demographic groups in Hartford, compared to Springfield. The comparison between the two cities suggests that more diverse demographic makeup across neighborhoods is associated with lessened vulnerability for single demographic groups and communities. While increased diversity does not address the risk to individuals, it can serve to reduce the stigmatization of already otherwise burdened communities.

Our data shows that Hispanic/Latino residents in both cities are the most vulnerable to higher concentrations of soil Pb (Figures 4c and 4f). For both cities, the soil Pb concentration was significantly higher where Hispanic/Latino populations made up a larger proportion of the census blocks (Figure S3 in Supporting Information S1). In Springfield, the distribution of housing age (Figure 3f) is almost identical to that of the Hispanic/Latino population (Figure S3h in Supporting Information S1) and that of Pb (Figure 2b), and somewhat similar to that of the distribution of first Quartile tax valuation (Figure S4c in Supporting Information S1) and multifamily housing (Figure 5e) indicating that residents reporting a Hispanic/Latino ethnicity tend to live in older, multi‐family housing.

Higher vulnerability to soil Pb for Hispanic/Latino communities has been observed in other cities (Del Rio, 2023; Masri et al., 2020), however we are not aware of other studies showing this for the Northeast region of the USA.

#### 4.2.2 City Specific Soil Pb Hazards for Children

Because they are in developmental stages, children are the most at risk when it comes to Pb contamination (Del Rio, M, 2023; Wani et al., 2015). The significantly higher Pb mean in blocks with a higher percentage of young children (Figure S4e in Supporting Information S1) suggests that the most vulnerable age group—babies and toddlers—are more at risk in these cities. Overall, both cities show that blocks with higher populations under 18 years of age have higher soil Pb than the blocks with fewer children (Figures S4g and S4h in Supporting Information S1). This observation is particularly important as Pb exposure, bioabsorption and bioretention are considerably greater in infants (Egendorf et al., 2020; Patriarca et al., 2000) with clear evidence that even limited exposure can lead to significant adverse neurological effects (Collin et al., 2022).

#### 4.2.3 Higher Vulnerability for Renters Than Owners

In both cities, the average Pb concentration in census blocks dominated by single family residential parcels was significantly lower than that in census blocks dominated by multi‐family residential parcels (Figures 5c and 5f). In the US, access to property is a desirable goal, inherent to the notion of fulfilling the so‐called “American Dream” (Goodman & Mayer, 2018). As such, owning a single‐family home is often viewed as an accomplishment. For this reason, the single‐family residential versus multi‐family residential difference reflects the dichotomy between owners and renters (An et al., 2022), which itself is correlated with economic status (Federal Reserve, 2025). Tax valuation of properties is also a marker of economic differences, in particular when compared with residential blocks. However, it must be kept in mind that, because of their size, multi‐family properties are typically taxed higher than single‐family homes. Overall, our data show that renters are more at risk of living in areas with elevated soil Pb, an observation in line with recent studies in other parts of the USA (Dietrich et al., 2023; Eisenberg et al., 2020; Masri et al., 2020). The opposite trend observed between the cities with respect to tax valuation can be explained by the difference in residential makeup of the cities. In Hartford, the majority of residential blocks are dominated by older buildings, and multi‐family blocks are typically taxed in a higher percentile group than single‐family residential blocks (Figure S15a in Supporting Information S1). In Springfield, the differences are less obvious due to a larger fraction of newer residential blocks, in particular as single‐family residences, and taxation rates being more widely spread (Figure S15b in Supporting Information S1). This suggests, similar to the trends observed for the demographic makeup of the cities (Section 4.2.1), that the higher economic diversity (i.e., lower segregation) observed in Springfield is associated with lessened vulnerability for single demographic groups and communities.

### 4.3 Intersection of Factors for City‐Specific Vulnerability Analyses

Our study of soil Pb in Hartford and Springfield revealed that different populations are at risk of living in elevated soil Pb areas. Taken separately, the populations most at risk in Hartford are those living in older housing, Hispanic/Latino residents, children, middle class (third quartile), and/or those living in multi‐family housing. In Springfield, the populations most at risk are those living in older housing, Hispanic/Latino, non‐Hispanic Black, children under 18 years, in the lower tax quartile, and/or those living in multi‐family housing. While there are similarities between the cities, the main difference concerns economic vulnerability, with the census blocks within the third tax valuation quartile more vulnerable in Hartford and those within the first quartile more vulnerable in Springfield (Figure S5 in Supporting Information S1).

**Figure 6:**
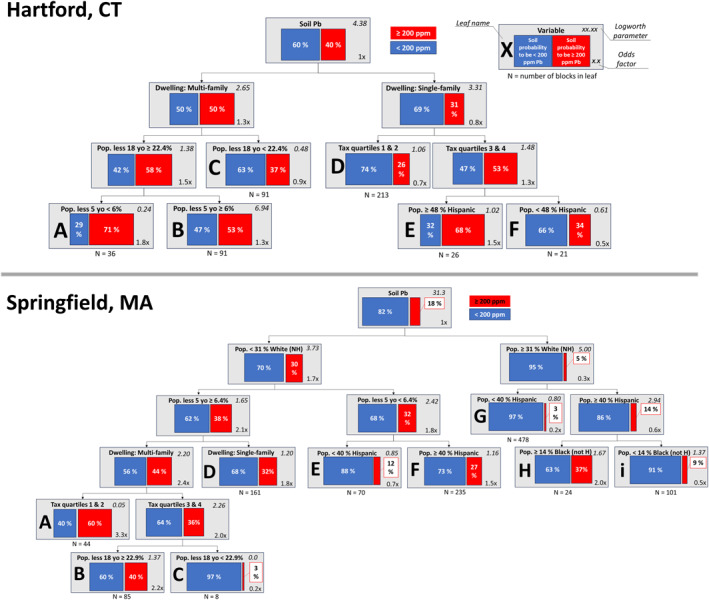
Decision tree partition model for above EPA screening level in residentially dominated census blocks. Factors affecting the likelihood of living in a contaminated area of the city are assessed on either side of the mean, with odds factors representing the increase in likelihood compared to the general population. The model was trained using 75% of the blocks and the number of splits limited to splits with a logworth value below 1.3. Details of the statistical procedure and fit are given in the Supporting Information S1.

Of course, demographic and economic factors overlap and intersect, meaning that the highest risk corresponds to residents belonging to several of these groups. In fact, soils have been shown to reflect not only pedogenetic processes but also a suite of socio‐historical factors, contributing to a “material politics of place” within the context of environmental justice (McClintock, 2015). These intersections are best represented shown in Figure 6, which represents the intersection of all factors (ethnicity, race, age, and housing characteristics) with soil Pb values in residential blocks only. To be able to best compare the two cities, we did not include the HOLC redlining parameter in Hartford. In Hartford, the likelihood for a block to have a mean Pb soil value above 200 ppm is 40%, more than double that of Springfield (18%). We stopped the splitting of the tree branches when the logworth value was below 1.3, corresponding to *α* = 0.05. Of note, due to a larger number of blocks, Springfield's model is more robust than Hartford's (Figure S16 in Supporting Information S1).

In Hartford, the most determining factor for soil Pb is the type of residential dwelling dominating the blocks, with soils from multi‐family dwellings corresponding to a higher likelihood of having above 200 ppm Pb (1.3 times the city likelihood) than in blocks dominated by single‐family dwellings (0.8 times the city likelihood). In the branch dominated by multi‐family dwellings, blocks with a percent youth (<18 yo) population above the city median are more likely than those below the median (1.5 times vs. 0.9 times, leaf C) to have soils with over 200 ppm Pb. Interestingly, within these blocks (multi‐family, above median youth population), the blocks with the highest odds to have soil Pb above 200 ppm are those with a population of children under 5 yo *below* the city median, corresponding to a 1.8 times increase in risk (leaf A) compared to the blocks with the highest population of children under 5 yo (1.3 times increase, leaf B). In the single‐family residences branch of the partition model, the next determining factor is taxation level with the blocks dominated by residences taxed the lowest being least likely to be above the EPA limit for Pb in soils (0.7 times the city odds, leaf D). The odds however increase to 1.3 times when the taxation level is higher and to 1.5 times for single‐family, higher taxation level blocks with a Hispanic/Latino population above the city median value (leaf E). On the other end, similar blocks with a Hispanic/Latino population below the city median are much less likely (0.5 times) to have high Pb in the soils than the city odds (Leaf F). Overall, the partition model for Hartford indicates that the most vulnerable population with respect to Pb in soil in the city is composed of children of all racial/ethnic groups living in multi‐family dwellings (leaves A and B). On the other end, the least impacted demographic with respect to Pb in soils in the city of Hartford is non‐Hispanic and lives in a single‐family dwelling of a higher taxation level (leaf F).

In Springfield, the most determining factor for soil Pb is demographic as blocks with more than 31% of the population identifying as non‐Hispanic White constitute the branch with the least probability for high Pb in soils. Further, within these blocks, blocks with less than 40% Hispanic/Latino population are 0.2 times less likely to be above the EPA limit than the city, leaf G). However, within this branch, blocks with over 14% non‐Hispanic Black or African American that also have over 40% Hispanic/Latino residents are twice as likely to have high Pb than the overall city likelihood (leaf F), while blocks with less than 14% non‐Hispanic Black or African American and over 40% Hispanic/Latino residents are 0.5 times as likely to have high soil Pb (leaf i). In the high likelihood branch, it appears that a higher population of children (under 5 yo) is the second most deterministic factor: blocks with a population of children under 5 yo higher than the city median are twice as likely to have high soil Pb as the city average. Within this branch, blocks dominated by multi‐family housing and lower taxation are the most likely to have high soil Pb, 3.3 times more than the city's likelihood (leaf A). For multi‐family dominated blocks in the higher taxation quartiles, the determining factor is population age, with blocks having fewer youth residents very unlikely to have high Pb in soils (leaf C) compared to the blocks with more than 22.9% residents under 18 yo (leaf B). In this branch, for blocks with a high population of children under 5 yo dominated by single‐family residences, the likelihood remains higher than that of the city at 1.8 times (leaf D). In blocks with a lower population of children under 5 yo, the deterministic factor is ethnicity as blocks with a Hispanic/Latino population below the city median are 0.7 times as likely (leaf E) to have high Pb in soil while those with a higher Hispanic/Latino population are 1.5 times as likely (leaf F), compared to the city odds. Overall, the partition model for Springfield indicates that the most vulnerable population with respect to Pb in soil in the city is composed of children living in multi‐family dwellings of lower taxation value in blocks with a lower than median proportion of non‐Hispanic White residents (leaf A). On the other hand, the least impacted demographic with respect to Pb in soils in the city of Springfield is non‐Hispanic Whites (leaf G).

Considering the intersection of these vulnerability factors is fundamental to better design approaches of mitigation, abatement, and remediation of Pb in soils. It prompts fresh policies and resource allocation to the most vulnerable groups. In both cities, youth populations and type of residential dwellings are important factors. Because children are the most vulnerable population with respect to Pb contamination, our models suggest that remediation efforts should first address blocks with a population of children above the city average that are also dominated by multi‐family residential parcels.

### 4.4 Soil Legacy

While it is established that the main source of Pb in urban soils is legacy Pb‐paint and gasoline, the link to vulnerabilities within population groups is less clear. A growing body of research has shown that structural racism has created long‐lasting environmental injustices in the USA (Locke et al., 2021; Pulido, 2017; Rothstein, 2017). This has been particularly studied with respect to soil contamination because the persistence of soil contaminants makes soil chemistry a reflection of past practices and activities (McClintock, 2015). Although our data do not allow us to decipher with certainty the reason why specific groups, such as children living in multi‐family residences in Hartford, or Hispanic and Black children living in low tax quartile multi‐family housing in Springfield, are the most co‐located with soil Pb, analysis of the distribution of soil Pb as a function of historic redlining in Hartford (Figure S7 in Supporting Information S1) shows without ambiguity that current Pb concentrations in soils reflect past discriminatory practices for housing. Indeed, the neighborhoods defined in the 1937 HOLC maps as “best” (A) and “still desirable” (B) show significantly less soil Pb than the neighborhoods defined as “definitely declining” (C) and “hazardous” (D). Further, when integrated in the partition model for Hartford, HOLC ratings became the dominating determining factor for Pb (Figure S17 in Supporting Information S1). Nearly 90 years have passed since these maps were in effect, yet the soils have kept—and, indeed, perpetuated—that memory. Despite efforts to curb segregation, such as the Fair Housing Act passed in 1968 (HUD, 2024), economically vulnerable and racially marginalized communities are still experiencing its effects due to racist policies and legal frameworks (Rothstein, 2017) that perpetuate the economic impoverishment and geographic marginalization of people of color, while simultaneously enabling non‐Hispanic White residents to access higher quality housing in safer areas (Pulido, 2017).

## 5 Conclusions and Implications

Our extensive data analysis of the intersection of soil Pb contamination with demographic, economic, and housing characteristics shows that socio‐economically vulnerable communities are carrying the heaviest burden of environmental contamination in the studied urban systems. Using about 150 samples and publicly available data, we created a clear picture of the communities at risk in our cities of interest. We show that children and residents of multi‐family housing are at high risk of contact with high soil Pb in both cities. We also show that non‐Hispanic White communities are the least at risk in Springfield and that historical redlining has a clear relation to soil Pb. Historically, industrial hubs like Hartford and Springfield have depended on relatively high immigration, both from the US South and internationally. However, legacies of discriminatory housing and job market practices have created and maintained a mutual, recursive stigmatization of people and their places, resulting in a lack of resources, remediation, and recognition for diverse communities, and magnifying their environmental vulnerabilities.

Beyond the city‐specific outcomes for Hartford and Springfield, our work presents a framework of analysis that can be reproduced elsewhere. Sampling of individual soils is a relatively easy task that can be performed by community groups and residents through locally organized programs (Landes et al., 2019; Perdrial et al., 2020; US EPA, 2021). Reproduction of the analytical framework presented in the present study can then provide a tool to advocate for focused resources for mitigation and eventual remediation. While extensive in nature, we postulate that the analysis we carried out here could be replicated at a limited cost in many urban systems by taking advantage of newly developed databases for soil contaminants, such as the MapMyEnvironment database (https://www.mapmyenvironment.com/ on which this data set is available), and community participation approaches or “citizen science” (Egendorf et al., 2021; Filippelli et al., 2024). In particular, we show that complex intersections can be simplified for targeting remediation efforts to specific blocks. Using this approach, local agencies, advocacy groups, and communities could gain the power to identify and address long‐lasting environmental inequities.

The integration of multiple parameters in our model is non‐exhaustive. In fact, many other parameters could, and arguably should, be integrated to fully constrain the parameters responsible for high Pb in the environments. For example, when available, historic traffic data, median income, and Pb piping distribution would strengthen the model. We also would recommend the use of our model in areas where BLL values are available. In this case, the decision tree partition model would start at the BLL, above and below the screening level for the area in interest, and directly address the underlying cause of elevated BLL.

## Conflict of Interest

The authors declare no conflicts of interest relevant to this study.

## Supporting information

Supporting Information S1

## Data Availability

Individual analyses of the soils are available for visualization on the MapMyEnvironment database (https://www.mapmyenvironment.com/) and in Sirkovich et al., 2023 (https://doi.org/10.1016/j.envpol.2023.122441). All census data were acquired on the census data portal of the United States Census Bureau (https://www.census.gov/).

## References

[gh270159-bib-0001] 16 CFR § 1303 . (1977). Ban of lead‐containing paint and certain consumer products bearing lead‐containing paint. 42 FR 44199. Retrieved from https://www.ecfr.gov/current/title‐16/part‐1303

[gh270159-bib-0002] 40 CFR § 80 . (1996). Prohibition on gasoline containing lead or lead additives for highway use. 61 FR 3832. Retrieved from https://www.govinfo.gov/content/pkg/FR‐1996‐02‐02/pdf/FR‐1996‐02‐02.pdf

[gh270159-bib-0003] ACS . (2021). American Community Survey data. Retrieved from https://www.census.gov/programs‐surveys/acs/data.html

[gh270159-bib-0004] Agency for Toxic Substances and Disease Registry (ATSDR) . (2020). Toxicologial profile for lead (pp. 1–583). U.S. Deparment of Health and Human Services. Retrieved from https://www.atsdr.cdc.gov/toxprofiles/tp13.pdf

[gh270159-bib-0005] Allison, J. , & Allison, T. (2005). Partition coefficients for metals in surface water, soil, and waste. US EPA report EPA/600/R‐05/074. Retrieved from https://cfpub.epa.gov/si/si_public_record_report.cfm?Lab=NERL&dirEntryId=135783

[gh270159-bib-0006] An, B. Y. , Bostic, R. W. , Jakabovics, A. , Orlando, A. W. , & Rodnyansky, S. (2022). Small and medium multifamily housing: Affordability and availability. Housing Studies, 37(7), 1274–1297. 10.1080/02673037.2020.1842339

[gh270159-bib-0007] Bower, J. A. , Lister, S. , Hazebrouck, G. , & Perdrial, N. (2017). Geospatial evaluation of lead bioaccessibility and distribution for site specific prediction of threshold limits. Environment and Pollution, 229, 290–299. 10.1016/j.envpol.2017.05.064 28601018

[gh270159-bib-0008] Bullard, R. D. (1993). The threat of environmental racism. Natural Resources & Environment, 7, 23–56.

[gh270159-bib-0009] Burgoon, D. A. , Brown, S. F. , & Menton, R. G. (1995). Literature review of sources of elevated soil‐lead concentrations. In S. D. Allen Iske & M. E. Beard (Eds.), Lead in paint, soil and dust: Health risks, exposure studies, control measures, measurement methods, and quality assurance (pp. 76–91). ASTM International.

[gh270159-bib-0010] Busterud, J. W. (2025). Residential lead directive for CERCLA sites and RCRA Hazardous Waste Cleanup Program facilities, memorandum (p. 6). Retrieved from https://semspub.epa.gov/work/HQ/100003761.pdf

[gh270159-bib-0011] CAFE . (2025). UMASS design center in Springfield, Center for agriculture, food and the environment, UMAss Amherts. Retrieved from https://designcenter.umass.edu/resources/gis‐maps

[gh270159-bib-0012] Cattle, J. A. , McBratney, A. B. , & Minasny, B. (2002). Kriging method evaluation for assessing the spatial distribution of urban soil lead contamination. Journal of Environmental Quality, 31(5), 1576–1588. 10.2134/jeq2002.1576 12371175

[gh270159-bib-0013] Cerling, T. E. , Fernandez, D. P. , & Smith, K. R. (2026). Lead in archived hair documents a decline in lead exposure to humans since the establishment of the US Environmental Protection Agency. Proceedings of the National Academy of Sciences, 123–6(6), e2525498123. 10.1073/pnas.2525498123 PMC12890779 41628336

[gh270159-bib-0014] Cheng, Z. , Paltseva, A. , Li, I. , Morin, T. , Huot, H. , Egendorf, S. , et al. (2015). Trace metal contamination in New York City garden soils. Soil Science, 180(4/5), 167–174. 10.1097/SS.0000000000000126

[gh270159-bib-0015] Clark, H. F. , Brabander, D. J. , & Erdil, R. M. (2006). Sources, sinks, and exposure pathways of lead in urban garden soil. Journal of Environmental Quality, 35(6), 2066–2074. 10.2134/jeq2005.0464 17071875

[gh270159-bib-0016] Clark, J. J. , & Knudsen, A. C. (2013). Extent, characterization, and sources of soil lead contamination in small‐urban residential neighborhoods. Journal of Environmental Quality, 42(5), 1498–1506. 10.2134/jeq2013.03.0100 24216427

[gh270159-bib-0017] Collin, M. S. , Venkatraman, S. K. , Vijayakumar, N. , Kanimozhi, V. , Arbaaz, S. M. , Stacey, R. G. S. , et al. (2022). Bioaccumulation of lead (Pb) and its effects on human: A review. Journal of Hazardous Materials Advances, 7, 100094. 10.1016/j.hazadv.2022.100094

[gh270159-bib-0018] Conrad, O. , Bechtel, B. , Bock, M. , Dietrich, H. , Fischer, E. , Gerlitz, L. , et al. (2015). System for automated geoscientific analyses (SAGA) v. 2.1.4. Geoscientific Model Development, 8(7), 1991–2007. 10.5194/gmd-8-1991-2015

[gh270159-bib-0019] CT DEEP . (2025). Connecticut Department of Energy & Environmental Protection GIS open data. Retrieved from https://deepmaps.ct.gov/

[gh270159-bib-0020] Datko‐Williams, L. , Wilkie, A. , & Richmond‐Bryant, J. (2014). Analysis of U.S. soil lead (Pb) studies from 1970 to 2012. Science of the Total Environment, 468–469, 854–863. 10.1016/j.scitotenv.2013.08.089 24076506

[gh270159-bib-0021] Del Rio, M. (2023). Factors influencing inequities in lead exposure in United States children: A systematic review. International Public Health Journal, 15, 263–281.

[gh270159-bib-0022] Dietrich, M. (2020). Using historical atmospheric pollution data to prioritize environmental sampling in urban areas. City and Environment Interactions, 6, 100042. 10.1016/j.cacint.2020.100042

[gh270159-bib-0023] Dietrich, M. , Wood, L. R. , Shukle, J. T. , Herrmann, A. , & Filippelli, G. M. (2023). Contributory science reveals insights into metal pollution trends across different households and environmental media. Environmental Research Letters, 18(3), 034013. 10.1088/1748-9326/acbaad

[gh270159-bib-0024] DTSC . (2020). Human health risk assessment (HHRA) note number 3, DTSC‐modified screening levels (DTSC‐SLs): California Department of Toxic Substances Control – Human and Ecological Risk Office (p. 50).

[gh270159-bib-0025] Egendorf, S. P. , Gailey, A. D. , Schachter, A. E. , & Mielke, H. W. (2020). Soil toxicants that potentially affect children’s health. Current Problems in Pediatric and Adolescent Health Care, 50(1), 100741. 10.1016/j.cppeds.2019.100741 31987768

[gh270159-bib-0026] Egendorf, S. P. , Groffman, P. , Cheng, Z. , Menser, M. , Mun, J. , & Mielke, H. (2021). Applying a novel systems approach to address systemic environmental injustices: Constructing soil for limiting the legacy of lead (Pb). Elementa: Science of the Anthropocene, 9(1), 00174. 10.1525/elementa.2020.00174

[gh270159-bib-0027] Eisenberg, A. , Seymour, E. , Hill, A. B. , & Akers, J. (2020). Toxic structures: Speculation and lead exposure in Detroit’s single‐family rental market. Health & Place, 64, 102390. 10.1016/j.healthplace.2020.102390 32838900

[gh270159-bib-0028] Ericson, B. , Hu, H. , Nash, E. , Ferraro, G. , Sinitsky, J. , & Taylor, M. P. (2021). Blood lead levels in low‐income and middle‐income countries: A systematic review. The Lancet Planetary Health, 5(3), e145–e153. 10.1016/S2542-5196(20)30278-3 33713615

[gh270159-bib-0029] ESRI . (2019). ArcGIS desktop: Release 10.8. Environmental Systems Research Institute.

[gh270159-bib-0030] European Food Safety Authority (EFSA) . (2013). Scientific opinion on lead in food. EFSA Journal, 8(4), 1570. 10.2903/j.efsa.2010.1570 PMC13129927 42077631

[gh270159-bib-0031] Federal Reserve . (2025). Report on the Economic Well‐Being of U.S. Households in 2024—May 2025—Housing. Board of Governors of the Federal Reserve System. Retrieved from https://www.federalreserve.gov/publications/2025‐economic‐well‐being‐of‐us‐households‐in‐2024‐housing.htm

[gh270159-bib-0032] Fewtrell, L. J. , Prüss‐Ustün, A. , Landrigan, P. , & Ayuso‐Mateos, J. L. (2004). Estimating the global burden of disease of mild mental retardation and cardiovascular diseases from environmental lead exposure. Environmental Research, 94(2), 120–133. 10.1016/s0013-9351(03)00132-4 14757375

[gh270159-bib-0033] Filippelli, G. M. , Dietrich, M. , Shukle, J. , Wood, L. , Margenot, A. , Egendorf, S. P. , & Mielke, H. W. (2024). One in four US households likely exceed new soil lead guidance levels. GeoHealth, 8(6), e2024GH001045. 10.1029/2024GH001045 PMC11184640 38895173

[gh270159-bib-0034] Filippelli, G. M. , Risch, M. , Laidlaw, M. A. S. , Nichols, D. E. , & Crewe, J. (2015). Geochemical legacies and the future health of cities: A tale of two neurotoxins in urban soils. Elementa: Science of the Anthropocene, 3, 000059. 10.12952/journal.elementa.000059

[gh270159-bib-0035] Frank, J. J. , Poulakos, A. G. , Tornero‐Velez, R. , & Xue, J. (2019). Systematic review and meta‐analyses of lead (Pb) concentrations in environmental media (soil, dust, water, food, and air) reported in the United States from 1996 to 2016. Science of the Total Environment, 694, 133489. 10.1016/j.scitotenv.2019.07.295 31756826 PMC6918466

[gh270159-bib-0036] Goodman, L. S. , & Mayer, C. (2018). Homeownership and the American dream. The Journal of Economic Perspectives, 32(1), 31–58. 10.1257/jep.32.1.31

[gh270159-bib-0037] Goovaerts, P. (1999). Geostatistics in soil science: State‐of‐the‐art and perspectives. Geoderma, 89(1–2), 1–45. 10.1016/S0016-7061(98)00078-0

[gh270159-bib-0038] Guinn, B. E. , Baumgartner, K. B. , Boone, S. D. , Gaskins, J. , Zhang, H. , & Zierold, K. M. (2024). Association between topsoil lead concentrations and the risk of violent crime. Environmental Justice, 17(2), 110–119. 10.1089/env.2022.0077

[gh270159-bib-0039] Henry, H. , Naujokas, M. F. , Attanayake, C. , Basta, N. T. , Cheng, Z. , Hettiarachchi, G. M. , et al. (2015). Bioavailability‐Based in situ remediation to meet future lead (Pb) standards in urban soils and gardens. Environmental Science & Technology, 49(15), 8948–8958. 10.1021/acs.est.5b01693 26140328

[gh270159-bib-0040] Hollander, M. , Wolfe, A. , & Chicken, E. (2015). The one‐way layout. In Nonparametric statistical methods (pp. 202–288). John Wiley & Sons, Ltd. 10.1002/9781119196037.ch6

[gh270159-bib-0041] HUD (US Department of Housing and Urban Development . (2024). Retrieved from https://web.archive.org/web/20241028004552/https:/www.hud.gov/program_offices/fair_housing_equal_opp/fair_housing_act_overview

[gh270159-bib-0042] JECFA ‐ Joint FAO/WHO Expert Committee on Food Additives . (2011). Safety evaluation of certain food additives and contaminants. Lead WHO Food Addit. Ser., 64, 381–497.

[gh270159-bib-0043] Lacerda, D. , Pestana, I. A. , dos Santos Vergilio, C. , & de Rezende, C. E. (2023). Global decrease in blood lead concentrations due to the removal of leaded gasoline. Chemosphere, 324, 138207. 10.1016/j.chemosphere.2023.138207 36822521

[gh270159-bib-0044] Laidlaw, M. A. S. , & Filippelli, G. M. (2008). Resuspension of urban soils as a persistent source of lead poisoning in children: A review and new directions. Applied Geochemistry, 23(8), 2021–2039. 10.1016/j.apgeochem.2008.05.009

[gh270159-bib-0045] Laidlaw, M. A. S. , Filippelli, G. M. , Brown, S. , Paz‐Ferreiro, J. , Reichman, S. M. , Netherway, P. , et al. (2017). Case studies and evidence‐based approaches to addressing urban soil lead contamination. Appl. Geochem., Urban Geochemistry, 83, 14–30. 10.1016/j.apgeochem.2017.02.015

[gh270159-bib-0046] Landes, F. C. , Inauen, J. , Ponce‐Canchihuamán, J. , Markowski, K. , Ellis, T. K. , & van Geen, A. (2019). Does involving parents in soil sampling identify causes of child exposure to lead? A case Study of community engagement in mining‐impacted towns in Peru. GeoHealth, 3(8), 218–236. 10.1029/2019GH000200 32159043 PMC7007120

[gh270159-bib-0047] Larsen, B. , & Sánchez‐Triana, E. (2023). Global health burden and cost of lead exposure in children and adults: A health impact and economic modelling analysis. The Lancet Planetary Health, 7(10), e831–e840. 10.1016/S2542-5196(23)00166-3 37714172

[gh270159-bib-0048] Locke, D. H. , Hall, B. , Grove, J. M. , Pickett, S. T. A. , Ogden, L. A. , Aoki, C. , et al. (2021). Residential housing segregation and urban tree canopy in 37 US Cities. Npj Urban Sustain, 1, 1–9. 10.1038/s42949-021-00022-0

[gh270159-bib-0049] Masri, S. , LeBrón, A. , Logue, M. , Valencia, E. , Ruiz, A. , Reyes, A. , et al. (2020). Social and spatial distribution of soil lead concentrations in the City of Santa Ana, California: Implications for health inequities. Science of the Total Environment, 743, 140764. 10.1016/j.scitotenv.2020.140764 32663692 PMC7492407

[gh270159-bib-0050] MassDEP . (2014). Similar soils provision guidance for identifying when soil concentrations at a receiving location are “Not Significantly Lower Than” managed soil concentrations pursuant to 310 CMR 40.0032(3). Massachusetts Department of Environmental Protection. Retrieved from https://www.mass.gov/info‐details/similar‐soils‐regulation‐policy

[gh270159-bib-0051] MassGIS . (2026). MassGIS (Bureau of Geographic Information). Website. Retrieved from https://www.mass.gov/orgs/massgis‐bureau‐of‐geographic‐information

[gh270159-bib-0052] McClintock, N. (2015). A critical physical geography of urban soil contamination. Geoforum, 65, 69–85. 10.1016/j.geoforum.2015.07.010

[gh270159-bib-0053] McFarland, M. J. , Hauer, M. E. , & Reuben, A. (2022). Half of US population exposed to adverse lead levels in early childhood. Proceedings of the National Academy of Sciences, 119(11), e2118631119. 10.1073/pnas.2118631119 PMC8931364 35254913

[gh270159-bib-0054] Mielke, H. W. , Anderson, J. C. , Berry, K. J. , Mielke, P. W. , Chaney, R. L. , & Leech, M. (1983). Lead concentrations in inner‐city soils as a factor in the child lead problem. American Journal of Public Health, 73(12), 1366–1369. 10.2105/ajph.73.12.1366 6638229 PMC1651267

[gh270159-bib-0055] Mielke, H. W. , Berry, K. J. , Mielke, P. W. , Powell, E. T. , & Gonzales, C. R. (2005). Multiple metal accumulation as a factor in learning achievement within various New Orleans elementary school communities. Environmental Research, 97(1), 67–75. 10.1016/j.envres.2004.01.011 15476735

[gh270159-bib-0056] Mielke, H. W. , Blake, B. , Burroughs, S. , & Hassinger, N. (1984). Urban lead levels in Minneapolis: The case of the Hmong children. Environmental Research, 34(1), 64–76. 10.1016/0013-9351(84)90076-8 6723610

[gh270159-bib-0057] MP Statistical Discovery LLC . (2022). JMP ®17 Automation Reference. JMP Statistical Discovery LLC.

[gh270159-bib-0058] Needleman, H. (2004). Lead poisoning. Annual Review of Medicine, 55(1), 209–222. 10.1146/annurev.med.55.091902.103653 14746518

[gh270159-bib-0059] Needleman, H. , Gunnoe, C. , Leviton, A. , Reed, R. , Peresie, H. , Maher, C. , & Barrett, P. (1979). Deficits in psychologic and classroom performance of children with elevated dentine lead levels. New England Journal of Medicine, 300(13), 689–695. 10.1056/nejm197903293001301 763299

[gh270159-bib-0060] Nelson, R. K. , Winling, L. , Marciano, R. , & Connolly, N. (n.d.). Mapping inequality. American Panorama. Retrieved from https://dsl.richmond.edu/panorama/redlining/

[gh270159-bib-0061] Open Data Hartford . (2026). Open data Hartford—Search, visualize, download, create. Website. Retrieved from https://data.hartford.gov/

[gh270159-bib-0062] O’Shea, M. J. , Krekeler, M. S. , Vann, D. R. , & Giere, R. (2021). Investigation of Pb‐contaminated soil and road dust in a polluted area of Philadelphia. Environmental Monitoring and Assessment, 193(7), 440. 10.1007/s10661-021-09213-9 34164717 PMC8415436

[gh270159-bib-0063] Patriarca, M. , Menditto, A. , Rossi, B. , Lyon, T. D. B. , & Fell, G. S. (2000). Environmental exposure to metals of newborns, infants and young children. Microchemical Journal, 67(1–3), 351–361. 10.1016/S0026-265X(00)00088-6

[gh270159-bib-0064] Perdrial, N. , Walser, S. , Massey, C. , & Bierman, P. (2020). Lead in water and soil: Education and assessment involving Vermont middle and high school students. Paper presented at the Goldschmidt, virtual. 10.46427/gold2020.2061

[gh270159-bib-0065] Pfeiffer, D. , Schafran, A. , & Wegmann, J. (2021). Vulnerability and opportunity: Making sense of the rise in single‐family rentals in US neighbourhoods. Housing Studies, 36(7), 1026–1046. 10.1080/02673037.2020.1739235

[gh270159-bib-0066] Pirkle, J. L. , Brody, D. J. , Gunter, E. W. , Kramer, R. A. , Paschal, D. C. , Flegal, K. M. , & Matte, T. D. (1994). The decline in blood lead levels in the United States: The National Health and Nutrition Examination Surveys (NHANES). Jama, 272(4), 284–291. 10.1001/jama.1994.03520040046039 8028141

[gh270159-bib-0067] Pulido, L. (2000). Rethinking environmental racism: White privilege and urban development in Southern California. Annals of the Association of American Geographers, 90(1), 12–40. 10.1111/0004-5608.00182

[gh270159-bib-0068] Pulido, L. (2017). Geographies of race and ethnicity II: Environmental racism, racial capitalism and state‐sanctioned violence. Progress in Human Geography, 41(4), 524–533. 10.1177/0309132516646495

[gh270159-bib-0069] QGIS.org . (2026). QGIS geographic information system. QGIS Association. 10.5281/zenodo.6139224

[gh270159-bib-0070] Reeder, R. J. , Schoonen, M. A. A. , & Lanzirotti, A. (2006). Metal speciation and its role in bioaccessibility and bioavailability. Reviews in Mineralogy and Geochemistry, 64(1), 59–113. 10.2138/rmg.2006.64.3

[gh270159-bib-0071] Resongles, E. , Dietze, V. , Green, D. C. , Harrison, R. M. , Ochoa‐Gonzalez, R. , Tremper, A. H. , et al. (2021). Strong evidence for the continued contribution of lead deposited during the 20th century to the atmospheric environment in London of today. Proceedings of the National Academy of Sciences, 118(26), e2102791118. 10.1073/pnas.2102791118 PMC8256035 34155116

[gh270159-bib-0072] Retief, F. P. , & Cilliers, L. (2005). Lead poisoning in ancient Rome. Acta Theologica, (7), 147–164. 10.38140/at.v0i7.2086

[gh270159-bib-0073] Rothstein, R. (2017). The color of law: A forgotten history of how our government segregated America. Liveright Publishing Corporation.

[gh270159-bib-0074] Sargent, J. D. , Brown, M. J. , Freeman, J. L. , Bailey, A. , Goodman, D. , & Freeman, D. H. (1995). Childhood lead poisoning in Massachusetts communities: Its association with sociodemographic and housing characteristics. American Journal of Public Health, 85(4), 528–534. 10.2105/AJPH.85.4.528 7702117 PMC1615119

[gh270159-bib-0075] Seaton, A. (2014). Saturnism: An old story of poisoning. QJM: International Journal of Medicine, 107–6(6), 501–502. 10.1093/qjmed/hcu070 24694547

[gh270159-bib-0076] Sirkovich, E. C. , Walser, S. L. , Perdrial, N. , & Richardson, J. B. (2023). Evaluating trace elements in urban forest soils across three contrasting New England USA towns and cities by pXRF and mass spectrometry. Environment and Pollution, 336, 122441. 10.1016/j.envpol.2023.122441 37652231

[gh270159-bib-0077] Solt, M. J. , Deocampo, D. M. , & Norris, M. (2015). Spatial distribution of lead in Sacramento, California, USA. International Journal of Environmental Research and Public Health, 12(3), 3174–3187. 10.3390/ijerph120303174 25789455 PMC4377958

[gh270159-bib-0078] Triantafilis, J. , Huckel, A. I. , & Odeh, I. O. A. (2001). Comparison of statistical prediction methods for estimating field‐scale clay content using different combinations of ancillary variables. Soil Science, 166(6), 415–427. 10.1097/00010694-200106000-00007

[gh270159-bib-0079] U.S. Census Bureau . (2023). American Community Survey (ACS). Retrieved from https://www.census.gov/programs‐surveys/acs/

[gh270159-bib-0080] U.S. Census Bureau . (2020a). Census of Population and Housing, updated every 10 years. Retrieved from https://www.census.gov/programs‐surveys/decennial‐census/decade.2020.html

[gh270159-bib-0081] U.S. Census Bureau . (2020b). Census redistricting data (P.L. 94‐171) shapefiles. Retrieved from https://www.census.gov/geographies/mapping‐files/time‐series/geo/tiger‐line‐file.2020.html#list‐tab‐790442341

[gh270159-bib-0082] US EPA . (2007). Standard operating procedure 4230.19A: Soil sampling at lead‐contaminated residential sites. Retrieved from https://semspub.epa.gov/work/07/30195438.pdf

[gh270159-bib-0083] US EPA, O . (2021). R1 success story: SoilSHOP, Portland, Maine. Retrieved from https://www.epa.gov/brownfields/r1‐success‐story‐soilshop‐portland‐maine

[gh270159-bib-0084] VDH . (2023). ENV/ECP general screening values soil (p. 26). Vermont Department of Health. Retrieved from www.healthvermont.gov/sites/default/files/documents/pdf/ENV_ECP_GeneralScreeningValues_Soil.pdf

[gh270159-bib-0085] Wade, A. M. , Richter, D. D. , Craft, C. B. , Bao, N. Y. , Heine, P. R. , Osteen, M. C. , & Tan, K. G. (2021). Urban‐soil pedogenesis drives contrasting legacies of lead from paint and gasoline in city soil. Environmental Science & Technology, 55(12), 7981–7989. 10.1021/acs.est.1c00546 34019756

[gh270159-bib-0086] Walser, S. L. , Sirkovich, E. C. , Richardson, J. B. , McStay, A. E. , & Perdrial, N. (2023). Moisture, organic matter, and large particle correction for accurate Pb portable X‐ray fluorescence assessment in urban soils. X‐Ray Spectrometry, 52(2), 72–82. 10.1002/xrs.3321

[gh270159-bib-0087] Wang, Z. , Wade, A. M. , Richter, D. D. , Stapleton, H. M. , Kaste, J. M. , & Vengosh, A. (2022). Legacy of anthropogenic lead in urban soils: Co‐occurrence with metal(loids) and fallout radionuclides, isotopic fingerprinting, and in vitro bioaccessibility. Science of the Total Environment, 806, 151276. 10.1016/j.scitotenv.2021.151276 34717995

[gh270159-bib-0088] Wani, A. L. , Ara, A. , & Usmani, J. A. (2015). Lead toxicity: A review. Interdisciplinary Toxicology, 8(2), 55–64. 10.1515/intox-2015-0009 27486361 PMC4961898

[gh270159-bib-0089] Yeter, D. , Banks, E. C. , & Aschner, M. (2020). Disparity in risk factor severity for early childhood blood lead among predominantly African‐American Black children: The 1999 to 2010 US NHANES. International Journal of Environmental Research and Public Health, 17(5), 1552. 10.3390/ijerph17051552 32121216 PMC7084658

[gh270159-bib-0090] 24 CFR § 982.53 . (n.d.). Equal opportunity requirements and protection for victims of domestic violence, dating violence, sexual assault, or stalking. Retrieved from https://www.law.cornell.edu/cfr/text/24/982.53

[gh270159-bib-0091] City of Springfield, MA . (2005). City of Springfield, MA—Analysis of impediments to fair housing. Fair Housing Planning. Retrieved from https://www.springfield‐ma.gov/planning/fileadmin/community_dev/Fair%20Housing%20AI%20FINAL.pdf

[gh270159-bib-0092] CTByTheNumbers.info . (2017). Housing stock in CT cities among nation’s oldest. Retrieved from https://ctbythenumbers.news/ctnews/2017/08/23/housing‐stock‐in‐ct‐cities‐among‐nations‐oldest

[gh270159-bib-0093] Love, W. D. (1914). The colonial history of Hartford: Gathered from the original records. Retrieved from https://www.google.com/url?sa=t&source=web&rct=j&opi=89978449&url=https://books.google.com/books%3Fid%3D‐aeAAAAAIAAJ%26printsec%3Dfrontcover%26hl%3Den

[gh270159-bib-0094] MADPH . (2021). 2021 annual childhood lead poisoning surveillance report. Massachusetts Department of Public Health. Retrieved from https://www.mass.gov/doc/2021‐annual‐childhood‐lead‐poisoning‐surveillance‐report‐0/download

[gh270159-bib-0095] Sassen, S. (1990). Economic restructuring and the American City. Annual Review of Sociology, 16(1), 465–490. 10.1146/annurev.so.16.080190.002341

[gh270159-bib-0096] Springfield, MA ‐ Our Plural History . (n.d.). Retrieved from https://ourpluralhistory.stcc.edu/firstpeoples/index.html

[gh270159-bib-0097] Springfield Office of Housing . (2018). Housing Study, City of Springfield, June 2018. Retrieved from https://www.springfield‐ma.gov/housing/fileadmin/housing/Housing_Study/Housing_Study_June_2018.pdf

[gh270159-bib-0098] Strahan, D. (2017). Lost Springfield. History Press Library Editions.

[gh270159-bib-0099] Walsh, A. (2013). Hartford: A global history. In X. Chen & N. Bacon (Eds.), Confronting urban legacy: Rediscovering Hartford and New England’s forgotten cities (pp. 21–45). Lexington Books.

