# Supplementary material for: Soil Lead Risks Associated With Urbanization Histories in Springfield MA and Hartford CT, USA
Source: Geohealth. 2026 Jun 15;10(6):e2026GH001854. doi: 10.1029/2026GH001854 (PMC13267425; doi:10.1029/2026GH001854)

GeoHealth

Supporting Information for

Soil Lead Risks Associated with Urbanization Histories in Springfield MA and Hartford CT, USA

N. Perdrial[1,2], S. L. Walser[1], E. C. Sirkovich[3], J. B. Richardson[3], †, M. Cope[1]

[1] University of Vermont, Geography and Geosciences, Burlington, VT, USA

[2] GUND Institute for the Environment, Burlington, VT, USA

[3] University of Massachusetts Amherst, Department of Geosciences, Amherst MA, 01003, USA

[4] University of Virginia, Department of Environmental Sciences, Charlottesville VA, 22901, US

Corresponding author: Nicolas Perdrial (nicolas.perdrial@uvm.edu)

† Address when work performed: University of Massachusetts Amherst, Department of Geosciences, Amherst MA, 01003, USA

Contents of this file

Introduction .... p2.
Urbanization histories .... p2.
Figure S1: Residential Security Map ratings .... p5.
Figure S2: Distribution of mean building construction dates .... p6.
Figure S3: Population density and demographics .... p7.
Figure S4: Percentage of residents below 5 years old and 18 years old .... p8.
Figure S5: Extrapolated soil Pb and tax valuation in residential census blocks .... p9.
Figure S6: Relationship between soil Pb and distance to nearest building and road .... p10.
Figure S7: Soil concentrations as a function of redlining grade in Hartford .... p11.
Statistical Procedure, Reports and Assessment. .... p12-13.
    Variance inflation factors (VIF) .... p12.
    Table S1: Results of the VIF analysis: .... p12.
    Steel-Dwass pairwise analysis .... p12.
    Decision tree protocol .... p13.
    Table S2: Partition model categorical variables cutoffs .... p13.
Figure S8. Pairwise Comparison for Figure 3d and 3h .... p14.
Figure S9. Pairwise Comparison for Figure 5a, 5b, 5c .... p15.
Figure S10. Pairwise Comparison for Figure 5d, 5e, 5f .... p16.
Figure S11. Pairwise Comparison for Figure 6c and 6f .... p17.
Figure S12. Pairwise Comparison for Figure S3e, S3f, S3g, S3h. .... p18-19.
Figure S13. Pairwise Comparison for Figure S4b and S4d .... p20.
Figure S14. Pairwise Comparison for Figure 10. .... P21.
Figure S15. Distribution of taxation quartiles .... p21.
Figure S16. Misclassification Rate .... p22.
Figure S17. Hartford partition model with HLOC rating .... p22.

Introduction

The figures and text contained in this supporting information are additional figures that the authors judge important but not necessary to understand the manuscript.

Tables are the statistical reports for all the Steel-Dwass tests performed in this study. They are provided for complete disclosure of the analyses performed.

Urbanization histories

Hartford, CT

Hartford is the state capital of Connecticut with a 2022 population of 121,054 (U.S. Census Bureau, 2020a). Appropriated from Indigenous peoples (mainly Pequot, Mohegan, and Narragansett) by English colonizers in 1635 (Love, 1914), the city is one of the oldest European settlements in the USA (Walsh, 2015). Initially a rural governmental and market center, Hartford developed into a mercantile center between 1740 and 1830, when its population more than doubled to about 9,000 inhabitants by 1820. The arrival of the railroad in 1839 triggered rapid economic development, fostering a change from a rural to an industrial center, attracting immigrants, particularly from Ireland, to reach about 30,000 inhabitants in 1860. Hartford became the sole state capital in 1874 developing distinct neighborhoods, including a downtown commercial and administrative area. In the early 20th century, Hartford developed into an important industrial center (Walsh, 2015), and its population boomed from immigration from eastern and southern Europe, reaching a peak of over 175,000 inhabitants in 1950.

The expansion of manufacturing in the early 20th century, including production of firearms, sewing machines, typewriters, and bicycles, resulted in a geographic shift from the Connecticut River banks in Downtown toward the south and east. A shift in housing also occurred in this era: as a result of the Federal Housing Act of 1934, homeownership was within reach for more families, but lending biases by the federal government (‘redlining’) and racist real-estate practices meant families of color were excluded from that opportunity (Rothstein, 2017), leading to a post-WWII national trend of white families moving to lower-density suburbs and spatial concentration of people of color in city centers. These processes dramatically transformed the demographics of the city from <10% non-white in 1950 to 50% in the 1970s and >70% in 2020 (Figure S1 from the Home Owners’ Loan Corporation’s (HOLC) ‘redlining’ maps - (Nelson et al., n.d.)). Today, Hartford’s population is 12.6% non-Hispanic White and 35.5% non-Hispanic Black or African American alone. Additionally, 44% of Hartford residents identify as Hispanic/Latino (US Census Bureau, 2020a). In keeping with post-war widespread American deindustrialization, the loss of manufacturing industries in Hartford resulted in a economic restructuring; the city fell from being one of the richest in the US in the 1950s to a 2021 poverty rate of 28.4%, double the national rate. This

resulted in 25.5% of houses within the city being owner-occupied, having a median value of $172,100, compared to the metro area's owner-occupied rate of 67% and median home value of $259,200 (U.S. Census Bureau. Prepared by Social Explorer, accessed Oct 30, 2023).

Approximately 66% of housing in the city of Hartford was built before 1960 (CTByTheNumbers.info, 2017). Older housing stock, particularly road-side wooden structures built before the 1978 ban on Pb paint, constitute the conditions most likely to result in Pb soil contamination. The State of Connecticut identified Hartford as one of five cities with the highest incidence of childhood Pb poisoning (24 CFR § 982.53, 2015).

Springfield, MA

Located 25 miles north of Hartford, the city of Springfield, Massachusetts was first settled by Europeans in the 17th century, who appropriated land from Indigenous Algonkian peoples (including Agawam and Nonotuck) ("Springfield, MA - Our Plural History," n.d.). Springfield flourished as an economic and industrial center in the Northeast USA in the 19th century and first half of the 20th century prior to the regional economic decline of the 1970s. First established as a trading post, the location of Springfield (between New York city and Boston, on the Connecticut River) and railroad development triggered its economic growth between the late 18th century (1,574 inhabitants in 1790) and the mid-19th century (>12,000 inhabitants in 1852 - Strahan, 2017). This economic prosperity attracted immigrants and developed residential areas. Continued growth stabilized Springfield as the economic and cultural center of western Massachusetts in the early 20th century when it had over 62,000 inhabitants. Similarly to Hartford's dynamic, Springfield experienced deindustrialization and economic restructuring after 1970: the city's factories were shuttered and the loss of tax revenue, along with racist housing practices (Rothstein, 2017), spurred 'white-flight' to outlying towns. The spatial concentration of poor communities of color within the city and broad disinvestment in city services continued the downward spiral (Sassen, 1990).

A fair housing report mandated in 2005 (City of Springfield, MA, 2005) identified evidence of discriminatory practices including redlining in the city. While no map of the New Deal-era's HOLC exist for Springfield, maps for nearby Chicopee and Holyoke were published in 1935 (Nelson et al., n.d.), undoubtedly extending to Springfield (Figure S1). Today, 26.3% of Springfield's inhabitants live in poverty and 47.3% of the homes are owner-occupied, with a median house value of $168,700. As assessed in 2018, only 14% of housing structures in Springfield were built since 1980 and "much of Springfield's housing, particularly in the lowest-income neighborhoods, is in poor condition" (Springfield Office of Housing, 2018). As highlighted in the 2021 annual childhood lead poisoning surveillance report from the Massachusetts Dept. of Public Health (MADPH, 2021), Springfield is one of 16 high-risk communities in the state by rate of incidence.

24 CFR § 982.53 - Equal opportunity requirements and protection for victims of domestic violence, dating violence, sexual assault, or stalking. [WWW Document], n.d. . LII Leg. Inf. Inst. URL https://www.law.cornell.edu/cfr/text/24/982.53 (accessed 10.30.23).

City of Springfield, MA, (2005), City of Springfield, MA - Analysis of impediments to fair housing. Fair Housing Planning. https://www.springfield-ma.gov/planning/fileadmin/community_dev/Fair%20Housing%20AI%20FINAL.pdf

CTByTheNumbers.info, 2017. Housing Stock in CT Cities Among Nation's Oldest [WWW Document]. Conn. Numbers. URL https://ctbythenumbers.news/ctnews/2017/08/23/housing-stock-in-ct-cities-among-nations-oldest (accessed 10.30.23).

Love W.D. (1914), The Colonial History of Hartford: Gathered from the Original Records.. Accessed from: https://www.google.com/url?sa=t&source=web&rct=j&opi=89978449&url=https://books.google.com/books%3Fid%3D-aeAAAAAIAAJ%26printsec%3Dfrontcover%26hl%3Den

MADPH (2021), 2021 Annual Childhood Lead Poisoning Surveillance Report. Massachusetts Department of Public Health. https://www.mass.gov/doc/2021-annual-childhood-lead-poisoning-surveillance-report-0/download

Nelson R.K., Winling L., Marciano R. & Connolly N. (n.d.), Mapping Inequality. Am. Panor. URL https://dsl.richmond.edu/panorama/redlining/

Rothstein R. (2017), The Color of Law: A Forgotten History of How Our Government Segregated America. Liveright Publishing Corporation, New York.

Sassen S. (1990), Economic Restructuring and the American City. *Annu. Rev. Sociol.* 16, 465–490. https://doi.org/10.1146/annurev.so.16.080190.002341

Springfield, MA - Our Plural History, n.d. https://ourpluralhistory.stcc.edu/firstpeoples/index.html (accessed 10.30.23).

Springfield Office of Housing, 2018. Housing Study, City of Springfield, June 2018. https://www.springfield-ma.gov/housing/fileadmin/housing/Housing_Study/Housing_Study_June_2018.pdf

Strahan D. (2017), Lost Springfield, Massachusetts. History Press Library Editions.

U.S. Census Bureau (2020a) Census of Population and Housing, updated every 10 years. https://www.census.gov/programs-surveys/decennial-census/decade.2020.html

Walsh A. (2013), Hartford: A Global History, in: Confronting Urban Legacy: Rediscovering Hartford and New England's Forgotten Cities. Eds: Chen X. and Bacon N., Lexington Books, 21-45.

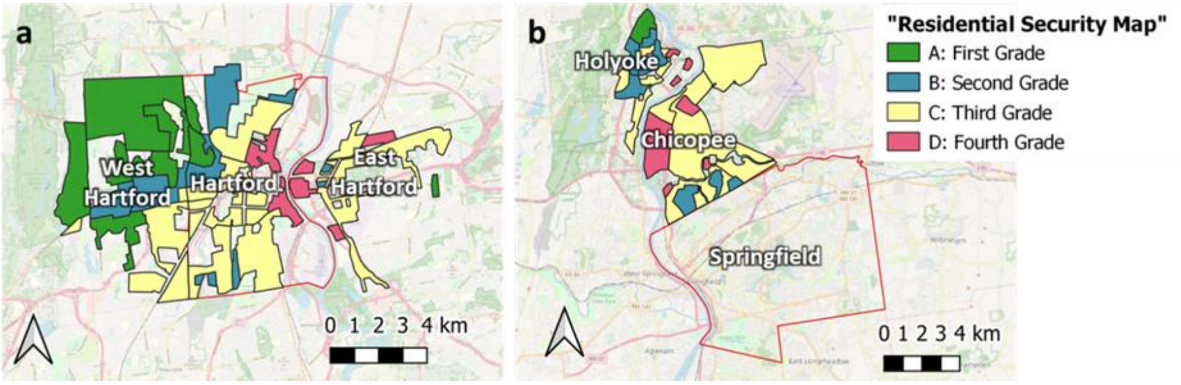

Figure S1. Residential security map (aka redlining maps) ratings for Hartford, CT (a) and in adjacent areas to Springfield, MA (b). Starting in the 1930s, the Home Owners' Loan Corporation - HOLC (a federal entity) assigned grades to residential neighborhoods reflecting their "mortgage security". An "A" grade represented minimal mortgage risks while "D" represented higher risks (deemed "hazardous"). The primary factor leading to lower grades was the presence or in-migration of people of color and immigrants, thereby creating a self-fulfilling prophecy of economic decline as banks refused to lend to those "hazardous" areas of the city. In effect the maps embodied and reinforced white supremacy culture by directing capital to native-born white families and away from African American and immigrant families. These practices were not made illegal until the Fair Housing Act of 1968. Data is from the Mapping Inequality database (Nelson et al., 2013). For more information on redlining visit https://dsl.richmond.edu/panorama/redlining/#loc=5/39.1/-94.58&text=intro

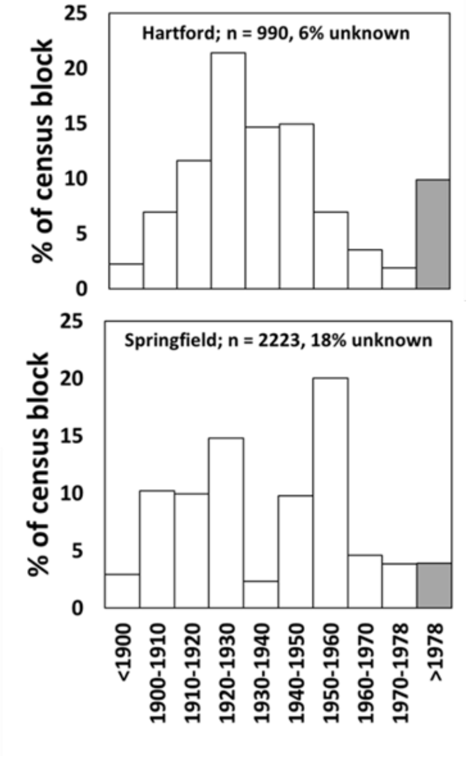

Figure S2. Distribution of mean building construction dates for both cities, excluding unknown blocks. Shading of the >1978 group represents blocks dominated by buildings built after the Pb-paint ban.

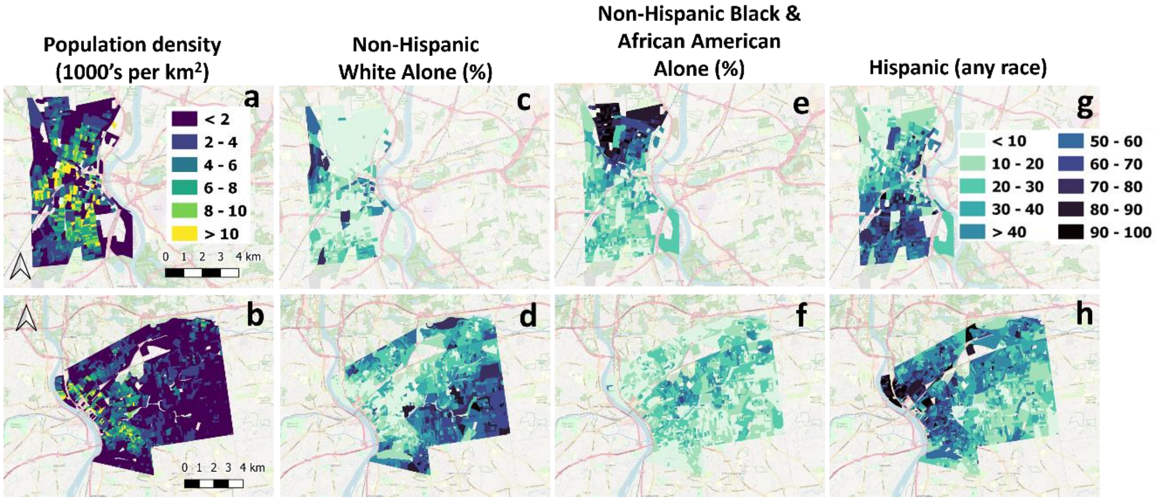

Figure S3. Population density in Hartford, CT (a) and Springfield, MA (b) in thousands per kilometer square; Percentage of the census block population identifying as White Alone, non-Hispanic in Hartford, CT (c) and Springfield, MA (d); Percentage of the census block population identifying as Black or African American Alone, non-Hispanic in Hartford, CT (e) and Springfield, MA (f); Percentage of the census block population identifying as Hispanic (of any race) in Hartford, CT (g) and Springfield, MA (h). Legend in a) applies to b) and Legend in g) applies to c), d), e), f), g) and h). The terms "White Alone", "Black or African-American Alone" and "Hispanic" are defined by the US Census Bureau, with the term "Alone" referring to respondents who claim only one racial category. This categorization is limited by the omission of non-Hispanic respondents who identify as more than one race.

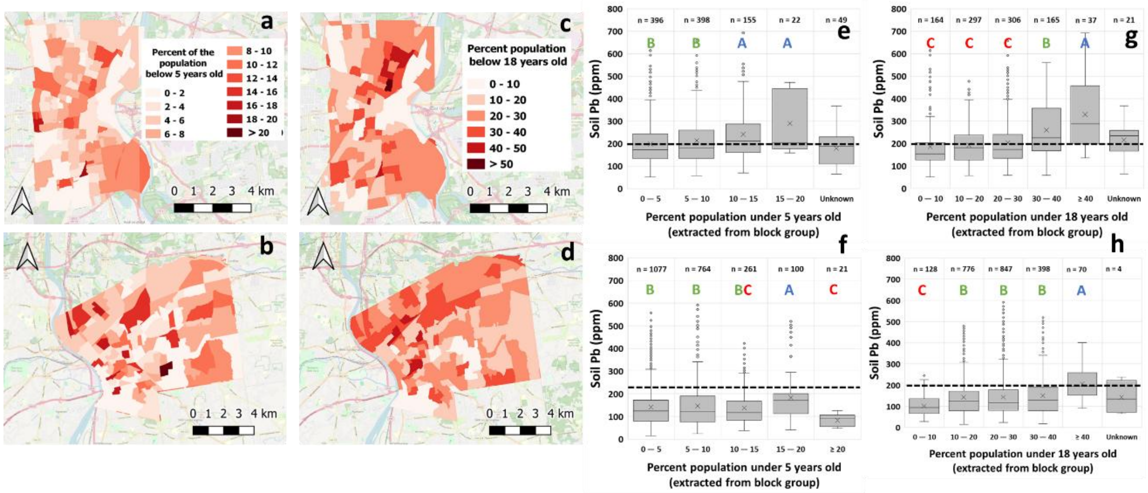

Figure S4. Visualization of the percentage of residents below 5 years old (a,b) and below 18 years old (c,d) in census blocks groups. Box-Plot and pair-wise analysis of the estimated soil Pb and child population was performed at the census block level by attributing the value of the block group to the blocks it contains. (e,f,g,h). Dotted lines in the box plots correspond to the EPA screening level for Pb in residential soils (200 ppm). Top row (a,c,e,g) corresponds to Hartford and bottom row (b,d,f,h) to Springfield.

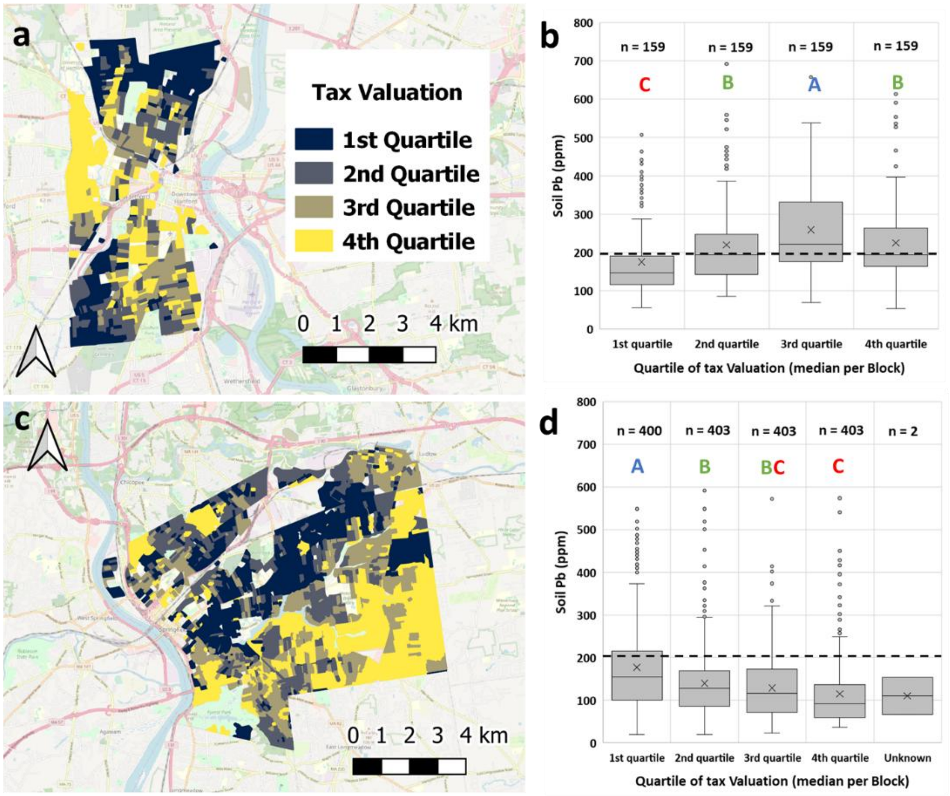

Figure S5. Correspondence between extrapolated soil Pb and tax valuation in residential census blocks for the cities of interest. Dotted lines in the box plots correspond to the EPA screening level for Pb in residential soils (200 ppm). Box plot characteristics are the same as Figure 3.

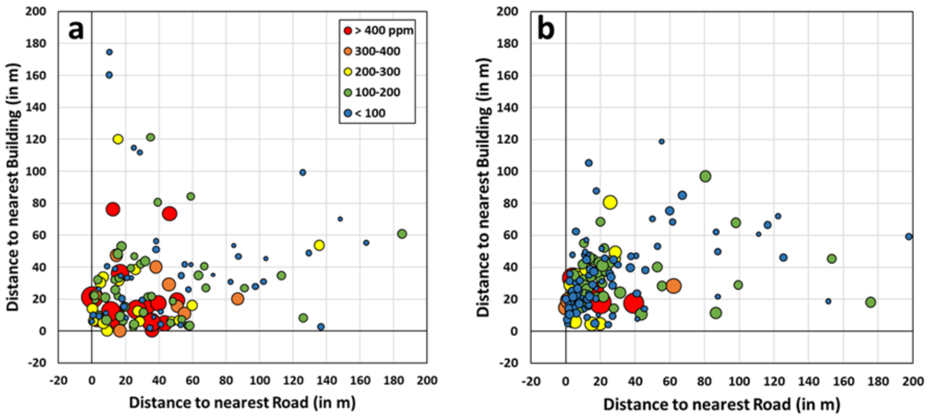

Figure S6. Relationship between soil Pb values and distance between soil sampling site and nearest building and road in Hartford (a) and Springfield (b). Both the size and color of the data points represent the soil Pb concentration analyzed by XRF. For reading convenience, one point located 256 m from a building and 12 m from a road, with a Pb concentration of 75 ppm has been omitted from b.

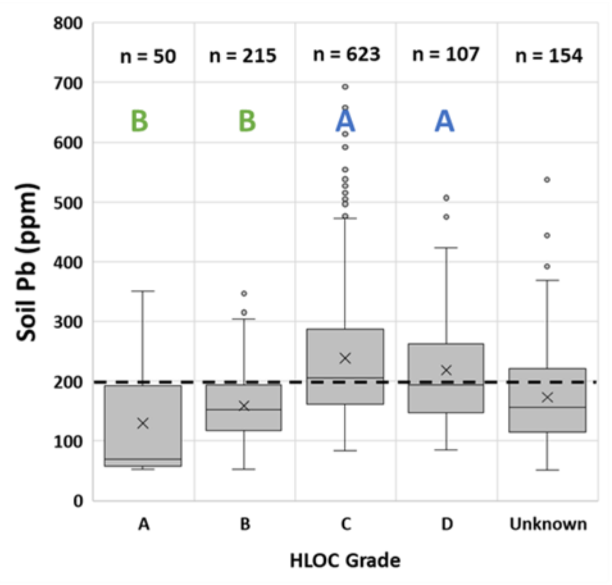

Figure S7. Soil concentrations as a function of redlining grade in Hartford (note that there is no redlining map for Springfield). HLOC stands for Home Owners' Loan Corporation. Dashed lines in the box plots correspond to the EPA screening value for Pb in residential soils (200 ppm). Box plot characteristics are the same as Figure 3.

Statistical Procedure, Reports and Assessment.

Variance inflation factors (VIF) were determined for each variable using a standard least square model response to the mean Pb values.

VIF is expressed as $VIF_{var} = \frac{1}{1 - RSquare_{X_{var}}}$

So that VIF is a measure of how much the standard error of the estimate $(1 - RSquare_{X_{var}})$ of the coefficient (X_{var}) is inflated due to multicollinearity.

According to JMP, the smallest possible value of VIF is 1.0, indicating a complete absence of multicollinearity, while VIF values greater than 5 or 10, or very high VIF values, indicate potential issues with multicollinearity https://www.jmp.com/en/statistics-knowledge-portal/what-is-multiple-regression/multicollinearity

Table S1: Results of the VIF analysis:

	Hartford	Springfield
Percent Black or African American (not Hispanic):	12.84	3.01
Percent Hispanic or latino:	10.89	6.24
Percent White (not Hispanic):	6.60	7.64
Population < 18 yo	1.68	1.46
Population < 5 yo	1.49	1.31
HLOC grade:	1.15	*NA*
Tax valuation:	1.14	1.02
Land-use Classification:	1.09	1.23
Construction date:	1.06	1.03

This analysis indicates, unsurprisingly, that demographic parameters present a significant collinearity. Accordingly, we performed our statistical analyses using a non-parametric model.

The **Steel-Dwass pairwise analysis** is the non-parametric equivalent to the Tukey-Kramer HSD test (honestly significant difference) test. To perform this test, we categorized each variable based on their values but irrespective of their distribution. In other words, except for tax valuation, we didn't force equal sample sizes in categories to preserve the natural skewness of the data. This is best exemplified by the categorization of the HLOC grade which are inherently skewed (see figure S6) yet must be interpreted accordingly. Categorization of the variables is given on their corresponding figures. The statistical analysis is a pairwise comparison of means and returns the following parameters:

Difference: Mean of the rank score of the observations in the first level minus the mean of the rank scores of the observations in the second level (-Level), where a continuity correction is applied.
Std Error Dif: The standard error of the Difference.
Z: The standardized test statistic, which has an asymptotic standard normal distribution under the null hypothesis of no difference in means.

Hodges-Lehmann: The Hodges-Lehmann estimator of the location shift. All paired differences consisting of observations in the first level minus observations in the second level are constructed. The Hodges-Lehmann estimator is the median of these differences.
Lower CL: The lower confidence limit for the Hodges-Lehmann statistic.
Upper CL: The upper confidence limit for the Hodges-Lehmann statistic.
p-Value: The p-value for the asymptotic Steel-Dwass test based on Z.

For each analysis we excluded the missing values. These are reported below the distribution graphs.
The connecting letter report represents significance between classes. In a given report, levels not connected by the same letter are significantly different. Variables are organized by decreasing Pb mean values in the connecting letter reports and increasing p-values in the differences reports.

Decision tree protocol

Each cities decision tree was performed on residential blocks only and categorical values. The model included all variables with categorical cutoff given in table S2 below.
The model was run in both cities using 75% of the blocks for training and 25% for validation corresponding to the split give in Table S2.
We split the trees using the logworth value as our significance indicator.
The logworth value is a transformed p-value, where a higher logworth value indicates a smaller p-value, and therefore a more significant effect or split. Here, we considered that A logworth value exceeding 1.3 was significant at the 0.05 level (because -log10(0.05) = 1.3)

Table S2: Partition model categorical variables cutoffs

	Hartford	Springfield
Number of residential blocks:		
Training	478	1206
Validation	159	402
Cutoff for Pb in soil (ppm)	200	200
Cutoff for Black or African American (not Hispanic), median (%)	25.4	14.3
Cutoff for Hispanic or latino, median (%)	48.0	40.0
Cutoff for White (not Hispanic), median (%)	6.7	30.9
Cutoff for Population < 18 yo, median (%)	22.4	22.9
Cutoff for Population < 5 yo, median (%)	6.0	6.4
Cutoff for HLOC grade	A&B vs C&D	*NA*
Cutoff for Tax valuation		
1st and 2nd quartiles	<$224,200	<$177,800
3rd and 4th quartiles	≥$224,200	≥$177,800
Cutoff for Land-use Classification	single- vs multi-family dwellings	
Cutoff for Construction date	before or after 1978	

***

Figure S8. Pairwise Comparison for Figure 3d and 3h

Hartford (CT) Building construction date pairwise comparisons of means using the Steel-Dwass test

Alpha-level set to 0.05

Connecting letters report

Level (date)	Levels not connected by same letter are significantly different.			Mean Pb (ppm)
<1950	A			220.33
> 1978	A	B		203.48
1950 - 1978		B		183.79

Ordered (by p-value) differences report

Level	- Level	Difference	Std Err Dif	Z	Hodges-Lehmann	Lower CL	Upper CL	p-Value
1950 - 1978	<1950	-82.5675	23.52509	-3.50976	-27.2018	-45.7482	-8.9843	0.0013
> 1978	1950 - 1978	14.4748	8.6579	1.67186	17.8578	-7.5066	45.8179	0.216
> 1978	<1950	-24.8176	25.17982	-0.98561	-9.2124	-31.4126	13.5063	0.586

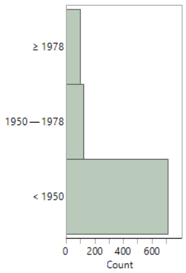

Distribution of values (missing data: 58)

Springfield (MA) Building construction date pairwise comparisons of means using the Steel-Dwass test

Alpha-level set to 0.05

Connecting letters report

Level (date)	Levels not connected by same letter are significantly different.			Mean Pb (ppm)
1850 - 1950	A			164.01
1950 - 1978		B		108.54
> 1978		B		106.53

Ordered (by p-value) differences report

Level	- Level	Difference	Std Err Dif	Z	Hodges-Lehmann	Lower CL	Upper CL	p-Value
1950 - 1978	1850 - 1950	-442.1020	25.6048	-17.2664	-51.278	-58.0754	-44.6327	<.0001
1850 - 1950	> 1978	-281.598	40.09259	-7.0237	-49.2875	-65.2745	-33.9261	<.0001
1950 - 1978	> 1978	5.164	23.78198	0.2171	1.0458	-10.4125	13.1366	0.9743

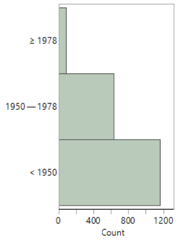

Distribution of values (Missing data: 341)

***

Figure S9. Pairwise Comparison for Figure 5a, 5b, 5c

Hartford (CT) White (NH) pairwise comparisons of means using the Steel-Dwass test

Alpha-level set to 0.05

Connecting letters report

Level (% in block)	Levels not connected by same letter are significantly different.			Mean Pb (ppm)
0 - 20	A			221.80
20 - 40	A			192.66
40 - 60	A			188.87
80 - 100	A			182.85
60 - 80	A			172.64

Ordered (by p-value) differences report

Level	- Level	Difference	Std Err Dif	Z	Hodges-Lehmann	Lower CL	Upper CL	p-Value
60 - 80	0 - 20	-77.7979	39.31585	-1.97879	-31.6211	-84.036	12.7937	0.2763
20 - 40	0 - 20	-41.6089	21.46197	-1.93873	-16.1325	-39.579	6.4372	0.2967
40 - 60	0 - 20	-41.8217	29.54574	-1.41549	-17.4137	-54.574	16.7382	0.6176
60 - 80	20 - 40	-8.3855	8.87244	-0.94512	-15.9166	-59.025	25.0105	0.8792
60 - 80	40 - 60	-3.4699	4.9562	-0.70011	-13.6041	-67.162	32.8704	0.9565
80 - 100	0 - 20	-61.0258	88.26406	-0.6914	-57.0415	-246.55	176.8562	0.9584
80 - 100	20 - 40	-3.4989	16.68004	-0.20976	-39.0480	-181.493	191.91	0.9996
80 - 100	40 - 60	-1.5029	7.16743	-0.20969	-36.6533	-203.361	207.1866	0.9996
40 - 60	20 - 40	-1.2323	7.92777	-0.15545	-1.8849	-36.364	34.4628	0.9999
80 - 100	60 - 80	0	4.15741	0	-1.4214	-203.186	220.2693	1

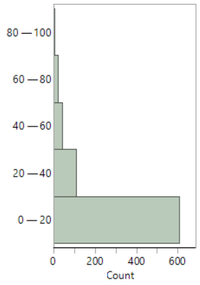

Distribution of values
(Missing Data: 207)

Hartford (CT) Black and African American (NH) pairwise comparisons of means using the Steel-Dwass test

Alpha-level set to 0.05

Connecting letters report

Level (% in block)	Levels not connected by same letter are significantly different.			Mean Pb (ppm)
40 - 60	A			244.52
0 - 20	A			223.90
20 - 40	A			222.48
60 - 80	A			214.12
80 - 100		B		153.66

Ordered (by p-value) differences report

Level	- Level	Difference	Std Err Dif	Z	Hodges-Lehmann	Lower CL	Upper CL	p-Value
80 - 100	20 - 40	-85.6724	11.3420	-7.5536	-57.2043	-79.861	-38.1903	<.0001
80 - 100	40 - 60	-55.1688	8.0269	-6.8730	-81.7097	-116.662	-52.9924	<.0001
80 - 100	0 - 20	-85.6966	13.1600	-6.5119	-51.4608	-75.434	-29.9187	<.0001
80 - 100	60 - 80	-34.9535	8.0653	-4.3338	-54.8254	-83.164	-22.4122	0.0001
40 - 60	0 - 20	32.2192	14.1629	2.2749	27.8995	-5.986	60.8459	0.153
40 - 60	20 - 40	22.9195	11.7746	1.9465	22.5598	-9.46	53.8584	0.2927
60 - 80	40 - 60	-13.3575	6.9549	-1.9206	-31.6705	-78.591	12.511	0.3062
20 - 40	0 - 20	11.5609	13.3886	0.8635	6.1721	-14.026	26.2307	0.9102
60 - 80	20 - 40	-6.9360	11.6830	-0.5937	-7.7380	-44.108	24.6332	0.9761
60 - 80	0 - 20	-4.5264	13.9992	-0.3233	-4.2344	-40.807	30.969	0.9976

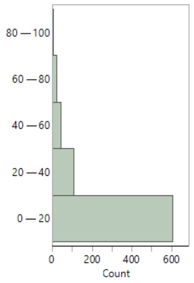

Distribution of values
(Missing Data: 207)

Hartford (CT) Hispanic (all) pairwise comparisons of means using the Steel-Dwass test

Alpha-level set to 0.05

Connecting letters report

Level (% in block)	Levels not connected by same letter are significantly different.			Mean Pb (ppm)
80 - 100	A	B		264.37
60 - 80	A			247.10
20 - 40	A	B		221.40
40 - 60		B		218.98
0 - 20			C	165.56

Ordered (by p-value) differences report

Level	- Level	Difference	Std Err Dif	Z	Hodges-Lehmann	Lower CL	Upper CL	p-Value
60 - 80	0 - 20	86.5938	11.7482	7.3708	63.5116	40.3008	90.1003	<.0001
40 - 60	0 - 20	80.2163	11.8040	6.7957	47.9088	29.3243	67.906	<.0001
20 - 40	0 - 20	69.1395	10.9410	6.3193	53.5065	31.497	74.0326	<.0001
80 - 100	0 - 20	53.5439	13.1129	4.0833	76.3877	25.5983	154.2798	0.0004
60 - 80	40 - 60	20.2398	11.7337	1.7249	14.8085	-8.5191	40.3277	0.4185
80 - 100	40 - 60	18.2751	13.0631	1.3990	24.2955	-24.4972	99.8048	0.6283
60 - 80	20 - 40	10.7748	10.8297	0.9949	11.0677	-17.5655	41.1314	0.8578
80 - 100	20 - 40	8.8668	9.1510	0.9690	22.4751	-31.6648	103.947	0.8692
40 - 60	20 - 40	-6.6126	10.9187	-0.6056	-5.4405	-27.8088	17.7729	0.9743
80 - 100	60 - 80	5.6152	12.8639	0.4365	8.9338	-45.9874	75.5021	0.9925

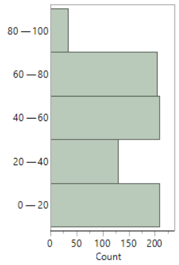

Distribution of values
(Missing Data: 207)

***

Figure S10. Pairwise Comparison for Figure 5d, 5e, 5f

Springfield (MA) White (NH) pairwise comparisons of means using the steel-Dwass test

Alpha-level set to 0.05

Connecting letters report

Level (% in block)	Levels not connected by same letter are significantly different.				Mean Pb (ppm)
0 - 20	A				185.39
20 - 40		B			141.54
40 - 60			C		109.23
60 - 80			C		91.97
80 - 100			C		89.88

Ordered (by p-value) differences report

Level	- Level	Difference	Std Err Dif	Z	Hodges-Lehmann	Lower CL	Upper CL	p-Value
60 - 80	20 - 40	-179.809	16.59089	-10.8378	-41.5361	-53.029	-31.4002	<.0001
80 - 100	0 - 20	-252.177	25.49229	-9.8923	-86.8731	-112.866	-64.6951	<.0001
40 - 60	0 - 20	-281.27	19.22617	-14.6296	-64.4211	-76.906	-52.8562	<.0001
60 - 80	0 - 20	-287.946	19.50712	-14.7611	-77.8236	-93.75	-63.3531	<.0001
40 - 60	20 - 40	-147.281	17.32639	-8.5004	-29.1028	-38.839	-20.0308	<.0001
80 - 100	20 - 40	-170.777	20.22367	-8.4444	-51.8230	-70.339	-36.2919	<.0001
20 - 40	0 - 20	-162.202	19.62576	-8.2648	-34.8469	-47.048	-23.3581	<.0001
80 - 100	40 - 60	-83.965	17.49936	-4.7982	-22.1369	-36.96	-9.7281	<.0001
60 - 80	40 - 60	-58.07	15.12299	-3.8399	-12.0721	-21.364	-3.5426	0.0012
80 - 100	60 - 80	-30.872	11.42459	-2.7022	-10.0449	-20.799	0.1065	0.0536

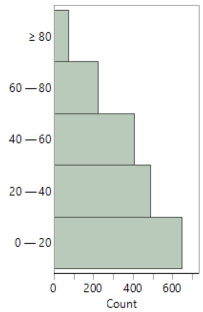

Distribution of values (Missing Data: 392)

Springfield (MA) Black and African American (NH) pairwise comparisons of means using the steel-Dwass test

Alpha-level set to 0.05

Connecting letters report

Level (% in block)	Levels not connected by same letter are significantly different.			Mean Pb (ppm)
40 - 60	A			176.57
20 - 40	A			156.77
60 - 80	A	B		155.13
0 - 20		B		132.36
80 - 100				none

Ordered (by p-value) differences report

Level	- Level	Difference	Std Err Dif	Z	Hodges-Lehmann	Lower CL	Upper CL	p-Value
20 - 40	0 - 20	161.47	26.25467	6.15016	20.9710	12.3435	29.58928	<.0001
40 - 60	0 - 20	224.563	38.22457	5.87484	42.5292	24.7614	60.99565	<.0001
40 - 60	20 - 40	51.479	18.61177	2.76592	20.9255	1.6758	41.07395	0.029
60 - 80	0 - 20	184.286	95.01718	1.93951	32.7771	-13.2979	75.36869	0.2115
60 - 80	20 - 40	27.721	40.16928	0.6901	12.3655	-39.0609	56.7422	0.9009
60 - 80	40 - 60	-6.092	10.04491	-0.60647	-10.6139	-72.4825	41.06983	0.9301

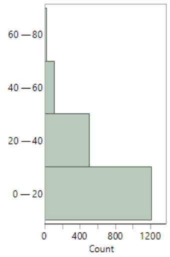

Distribution of values (Missing Data: 392)

Springfield (MA) Hispanic and Latino pairwise comparisons of means using the steel-Dwass test

Alpha-level set to 0.05

Connecting letters report

Level (% in block)	Levels not connected by same letter are significantly different.				Mean Pb (ppm)
60 - 80	A				197.97
80 - 100	A				185.40
40 - 60		B			157.89
20 - 40			C		120.64
0 - 20				D	92.30

Ordered (by p-value) differences report

Level	- Level	Difference	Std Err Dif	Z	Hodges-Lehmann	Lower CL	Upper CL	p-Value
40 - 60	0 - 20	257.57	17.5972	14.637	54.1962	44.478	64.3718	<.0001
60 - 80	0 - 20	214.524	14.27324	15.0298	89.7051	74.0898	106.3563	<.0001
60 - 80	20 - 40	207.792	17.86033	11.6343	64.5940	49.8865	80.2945	<.0001
40 - 60	20 - 40	165.357	19.20561	8.6098	30.3656	20.9102	39.8341	<.0001
80 - 100	0 - 20	137.791	14.03485	9.8178	84.1780	62.7549	107.7533	<.0001
20 - 40	0 - 20	143.497	17.94148	7.9981	23.5465	15.4484	32.2644	<.0001
80 - 100	20 - 40	140.261	19.63161	7.1447	58.2652	36.9712	79.9518	<.0001
60 - 80	40 - 60	101.68	17.44433	5.8288	33.5950	17.7713	49.9233	<.0001
80 - 100	40 - 60	59.517	19.00311	3.132	25.8405	3.4268	49.274	0.015
80 - 100	60 - 80	-11.019	12.0865	-0.9117	-9.9006	-38.9627	17.8849	0.8925

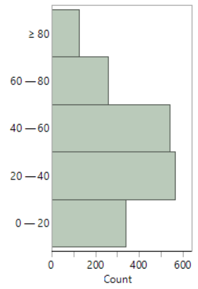

Distribution of values (Missing Data: 392)

***

Figure S11. Pairwise Comparison for Figure 6c and 6f

Hartford (CT) Landuse pairwise comparisons of means using the Steel-Dwass test

Alpha-level set to 0.05

Connecting letters report

Level	Levels not connected by same letter are significantly different.			Mean Pb (ppm)
Land/Park	A	B	C	258.45
Multi-Family	A			256.71
Commercial		B		219.02
Industrial	A	B	C	208.12
Single Family			C	185.77
Other/Unknown			C	181.11

Ordered (by p-value) differences report

Level	- Level	Difference	Std Err Dif	Z	Hodges-Lehmann	Lower CL	Upper CL	p-Value
Single Family	Multi-Family	-116.691	14.59823	-7.99349	-52.9462	-72.305	-34.898	<.0001
Other/Unknown	Multi-Family	-98.993	13.31982	-7.43203	-59.1201	-83.041	-36.513	<.0001
Other/Unknown	Commercial	-34.021	10.86168	-3.13225	-31.3992	-61.275	-2.573	0.0215
Single Family	Commercial	-42.335	14.11319	-2.99971	-26.2487	-51.634	-1.167	0.0323
Multi-Family	Commercial	36.699	13.3393	2.75118	27.9578	-0.975	57.524	0.0656
Other/Unknown	Land/Park	-47.54	18.98079	-2.50462	-62.6286	-156.413	10.195	0.1226
Single Family	Land/Park	-70.839	29.30581	-2.41723	-54.59	-140.559	11.655	0.1502
Multi-Family	Industrial	36.931	20.10661	1.83678	35.0942	-21.087	94.133	0.4419
Other/Unknown	Industrial	-22.413	14.52762	-1.54277	-26.2045	-81.318	25.719	0.6364
Land/Park	Industrial	4.688	3.72492	1.25842	42.8616	-83.882	157.034	0.8076
Land/Park	Commercial	13.194	10.58475	1.24655	28.238	-54.955	127.981	0.8138
Single Family	Industrial	-25.797	21.81179	-1.1827	-20.4515	-71.036	26.382	0.8453
Single Family	Other/Unknown	10.202	13.82017	0.73819	4.8806	-14.026	24.279	0.9772
Industrial	Commercial	-2.164	8.63161	-0.25075	-5.6682	-65.348	53.48	0.9999
Multi-Family	Land/Park	-3.075	26.89049	-0.11436	-3.453	-88.791	84.15	1

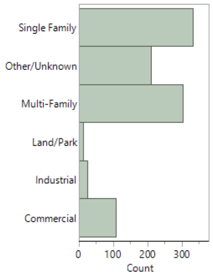

Distribution of values

Springfield (MA) Landuse pairwise comparisons of means using the steel-Dwass test

Alpha-level set to 0.05

Connecting letters report

Level	Levels not connected by same letter are significantly different.			Mean Pb (ppm)
Multi-Family	A			180.33
Other/Unknown		B		155.53
Commercial	A	B		154.77
Industrial	A	B	C	143.66
Land/Park	A	B	C	139.94
Single Family			C	127.94

Ordered (by p-value) differences report

Level	- Level	Difference	Std Err Dif	Z	Hodges-Lehmann	Lower CL	Upper CL	p-Value
Single Family	Multi-Family	-335.782	27.5638	-12.182	-50.5445	-62.1013	-39.332	<.0001
Single Family	Other/Unknown	-168.963	26.8215	-6.2995	-24.2846	-35.3739	-13.337	<.0001
Other/Unknown	Multi-Family	-81.073	16.6785	-4.8609	-26.7256	-42.2735	-11.215	<.0001
Single Family	Commercial	-178.038	38.6528	-4.6061	-27.7798	-45.5382	-11.169	<.0001
Multi-Family	Commercial	45.364	14.9893	3.0264	23.4626	1.2877	45.235	0.0298
Multi-Family	Land/Park	54.161	19.9948	2.7088	29.7546	-1.7186	64.391	0.0735
Single Family	Land/Park	-145.226	60.7817	-2.3893	-21.8718	-47.4783	4.626	0.1599
Multi-Family	Industrial	43.398	35.0184	1.2393	28.3159	-36.1245	102.885	0.8175
Single Family	Industrial	-137.853	114.6139	-1.2028	-24.3877	-78.7274	37.978	0.8357
Land/Park	Commercial	-5.67	8.1113	-0.699	-6.8464	-38.3231	22.541	0.9821
Other/Unknown	Commercial	-8.105	17.3389	-0.4674	-3.505	-25.1454	18.741	0.9972
Other/Unknown	Land/Park	7.626	24.1026	0.3164	3.5934	-28.3368	37.606	0.9996
Industrial	Commercial	-3.216	11.5801	-0.2777	-4.4747	-69.9211	59.619	0.9998
Land/Park	Industrial	-1.074	4.9757	-0.2158	-5.3426	-64.1058	60.233	0.9999
Other/Unknown	Industrial	3.321	43.0498	0.0771	1.5961	-63.9528	77.373	1

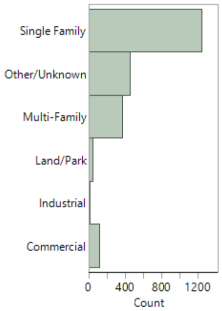

Distribution of values

***

Figure S12. Pairwise Comparison for Figure S3e, S3f, S3g, S3h.

Hartford (CT) children below 5 yo pairwise comparisons of means using the Steel-Dwass test

Alpha-level set to 0.05

Connecting letters report

Level (%<5yo)	Levels not connected by same letter are significantly different.			Mean Pb (ppm)
15 - 20	A			289.45
10 - 15	A			241.13
5 - 10	B			213.32
0 - 5	B			198.30
> 20				none

Ordered (by p-value) differences report

Level	- Level	Difference	Std Err Dif	Z	Hodges-Lehmann	Lower CL	Upper CL	p-Value
10 - 15	0 — 5	70.70628	15.08402	4.687496	37.08259	17.5907	58.2059	<.0001
10 - 15	5 - 10	54.13839	14.47061	3.741264	31.77935	10.3079	53.8551	0.0011
15 - 20	0 — 5	88.69066	26.46267	3.351538	61.36697	15.7771	163.694	0.0045
15 - 20	5 - 10	75.84671	24.74156	3.065559	53.99576	9.0925	126.8314	0.0117
15 - 20	10 - 15	15.80543	11.67389	1.353913	26.71996	-27.6066	125.7249	0.5285
5 - 10	0 - 5	15.08046	15.97945	0.943741	5.5445	-9.6541	20.9294	0.7812

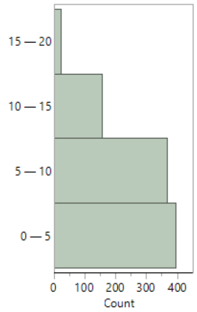

Distribution of values
(Missing Data: 49)

Springfield (MA) children below 5 yo pairwise comparisons of means using the steel-Dwass test

Alpha-level set to 0.05

Connecting letters report

Level (%<5yo)	Levels not connected by same letter are significantly different.			Mean Pb (ppm)
15 - 20	A			182.99
5 - 10		B		145.94
0 - 5		B		141.53
10 - 15		B	C	137.55
> 20			C	82.93

Ordered (by p-value) differences report

Level	- Level	Difference	Std Err Dif	Z	Hodges-Lehmann	Lower CL	Upper CL	p-Value
15 - 20	5 - 10	-45.951	8.41909	-5.45798	-78.2251	-115.594	-42.3909	<.0001
15 - 20	0 - 5	170.457	35.53453	4.79695	36.0604	16.176	55.4266	<.0001
> 20	15 - 20	58.486	12.273	4.76543	37.188	16.086	59.9164	<.0001
15 - 20	10 - 15	109.159	26.539	4.11316	33.1434	11.775	54.1202	0.0004
> 20	0 - 5	-260.215	69.87037	-3.72425	-40.2932	-75.294	-11.1488	0.0018
> 20	10 - 15	-67.606	18.49787	-3.65479	-37.3895	-70.056	-8.7603	0.0024
> 20	5 - 10	-176.214	50.15728	-3.51323	-40.9103	-85.66	-8.1899	0.004
10 - 15	0 - 5	16.294	25.1451	0.648	2.1571	-6.723	11.1602	0.9671
10 - 15	5 - 10	-11.095	21.22462	-0.52276	-2.4597	-15.568	10.0399	0.9851
5 - 10	0 - 5	-11.819	26.65801	-0.44335	-1.9233	-13.746	10.048	0.992

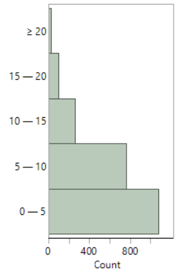

Distribution of values
(Missing Data: 0)

Hartford (CT) Youth (<18 yo) pairwise comparisons of means using the steel-Dwass test

Alpha-level set to 0.05

Connecting letters report

Level (%<18yo)	Levels not connected by same letter are significantly different.			Mean Pb (ppm)
> 40	A			329.43
30 - 40		B		259.34
20 - 30			C	203.43
10 - 20			C	192.69
0 - 10			C	186.12

Ordered (by p-value) differences report

Level	- Level	Difference	Std Err Dif	Z	Hodges-Lehmann	Lower CL	Upper CL	p-Value
30 - 40	0 - 10	71.94595	10.48814	6.859746	64.7874	38.8931	94.8416	<.0001
> 40	0 - 10	67.2926	10.58664	6.356367	123.9404	67.8472	217.7425	<.0001
> 40	10 - 20	99.05415	16.83446	5.884011	119.7977	63.0806	198.6007	<.0001
30 - 40	10 - 20	76.23165	12.96348	5.880492	55.3782	30.2806	82.4443	<.0001
> 40	20 - 30	98.77685	17.25922	5.723136	107.5138	55.6412	192.5224	<.0001
30 - 40	20 - 30	74.65196	13.14615	5.678618	48.8636	25.6682	74.8962	<.0001
> 40	30 - 40	31.53251	10.63325	2.965464	58.5371	4.8039	124.3728	0.0252
20 - 30	0 - 10	33.13995	13.14423	2.521255	16.113	-1.3006	34.5055	0.0859
10 - 20	0 - 10	28.63504	12.96078	2.209361	16.5479	-3.7825	36.4328	0.1761
20 - 30	10 - 20	4.34259	14.19078	0.306015	2.0115	-15.8165	20.175	0.9981

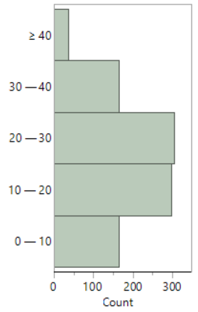

Distribution of values
(Missing Data: 21)

Springfield (MA) Youth (<18 yo) pairwise comparisons of means using the steel-Dwass test

Alpha-level set to 0.05

Connecting letters report

Level (%<18yo)	Levels not connected by same letter are significantly different.			Mean Pb (ppm)
> 40	A			206.68
30 - 40		B		146.86
20 - 30		B		143.30
10 - 20		B		142.78
0 - 10			C	101.66

Ordered (by p-value) differences report

Level	- Level	Difference	Std Err Dif	Z	Hodges-Lehmann	Lower CL	Upper CL	p-Value
> 40	0 - 10	79.41	8.51819	9.3224	102.484	77.6554	127.4607	<.0001
> 40	20 - 30	243.742	32.93892	7.39982	73.891	49.7836	97.3292	<.0001
> 40	10 - 20	219.233	30.49591	7.18892	72.503	47.4285	97.1941	<.0001
> 40	30 - 40	106.509	17.52876	6.07627	66.871	38.8005	92.9837	<.0001
30 - 40	0 - 10	82.399	15.44379	5.33542	33.077	16.106	51.83	<.0001
10 - 20	0 - 10	125.464	24.90957	5.03678	26.801	12.0865	43.1107	<.0001
20 - 30	0 - 10	132.177	26.70498	4.94951	25.403	11.3817	40.3011	<.0001
30 - 40	20 - 30	32.24	21.85015	1.47549	6.285	-5.1699	17.959	0.5785
30 - 40	10 - 20	26.344	20.90374	1.26027	5.558	-6.6499	17.616	0.7156
20 - 30	10 - 20	-6.402	23.28887	-0.27488	-0.939	-9.9636	7.9916	0.9988

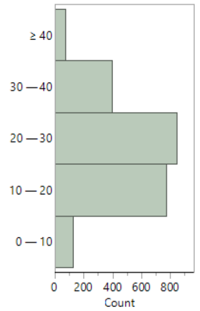

Distribution of values
(Missing Data: 4)

***

Figure S13. Pairwise Comparison for Figure S4b and S4d.

Hartford (CT) tax valuation (residential parcels only) pairwise comparisons of means using the steel-Dwass test

Alpha-level set to 0.05

Connecting letters report

Level	Levels not connected by same letter are significantly different.			Mean Pb (ppm)
3rd quartile	A			254.10
2nd quartile		B		226.54
4th quartile		B		213.04
1st quartile			C	173.40

Ordered (by p-value) differences report

Level	- Level	Difference	Std Err Dif	Z	Hodges-Lehmann	Lower CL	Upper CL	p-Value
3rd quartile	1st quartile	89.824	11.05046	8.12853	68.0428	49.0851	87.92459	<.0001
2nd quartile	1st quartile	69.6298	11.20912	6.21189	47.2455	28.3591	67.98446	<.0001
4th quartile	1st quartile	45.5492	9.77041	4.66196	50.4107	24.9743	74.8541	<.0001
3rd quartile	2nd quartile	29.057	11.57875	2.50951	22.7864	-0.6599	45.12507	0.0584
4th quartile	3rd quartile	-21.6229	10.72861	-2.01544	-21.6641	-54.8712	6.57969	0.1822
4th quartile	2nd quartile	1.2591	11.02871	0.11417	1.2665	-29.2594	29.03555	0.9995

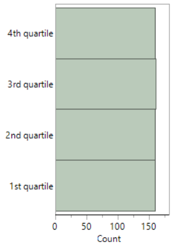

Distribution of values (Missing Data: 0)

Springfield (MA) tax valuation (residential parcels only) pairwise comparisons of means using the steel-Dwass test

Alpha-level set to 0.05

Connecting letters report

Level	Levels not connected by same letter are significantly different.			Mean Pb (ppm)
1st quartile	A			177.00
2nd quartile		B		139.32
3rd quartile		B	C	128.94
4th quartile			C	114.73

Ordered (by p-value) differences report

Level	- Level	Difference	Std Err Dif	Z	Hodges-Lehmann	Lower CL	Upper CL	p-Value
4th quartile	1st quartile	-172.402	16.35034	-10.5443	-49.7451	-61.9903	-38.4107	<.0001
4th quartile	2nd quartile	-114.279	16.38093	-6.9764	-27.3524	-36.8931	-18.0834	<.0001
3rd quartile	1st quartile	-112.761	16.36057	-6.8923	-35.0471	-47.971	-22.259	<.0001
2nd quartile	1st quartile	-79.76	16.37082	-4.8721	-24.1324	-36.9905	-11.3207	<.0001
4th quartile	3rd quartile	-68.795	16.37072	-4.2023	-14.4832	-24.1998	-5.7352	0.0002
3rd quartile	2nd quartile	-43.709	16.39107	-2.6667	-11.3594	-22.0081	-0.4529	0.0384

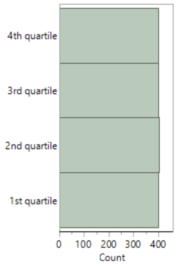

Distribution of values (Missing Data: 2)

Figure S14. Pairwise Comparison for Figure 10.

Hartford (CT) HLOC pairwise comparisons of means using the steel-Dwass test

Alpha-level set to 0.05

Connecting letters report

Level ("grade")	Levels not connected by same letter are significantly different.			Mean Pb (ppm)
C	A			243.10
D	A			216.18
B		B		150.98
A			C	136.28

Ordered (by p-value) differences report

Level	- Level	Difference	Std Err Dif	Z	Hodges-Lehmann	Lower CL	Upper CL	p-Value
C	B	190.566	18.11458	10.5201	70.322	53.0931	88.7987	<.0001
D	B	56.702	9.87462	5.7422	49.284	26.9974	73.5265	<.0001
C	A	155.741	34.93427	4.4581	104.211	56.2629	150.4101	<.0001
D	A	28.537	8.00843	3.5634	83.267	38.2312	126.3993	0.0021
D	C	-46.415	20.49751	-2.2644	-20.583	-44.0536	2.8038	0.1064
B	A	24.375	12.52979	1.9454	35	-14.6434	72.0577	0.2092

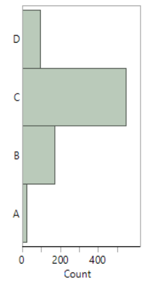

Distribution of values

***

Figure S15. Distribution of taxation quartiles between multi- and single-family dominated residential blocks as a function of construction dates.

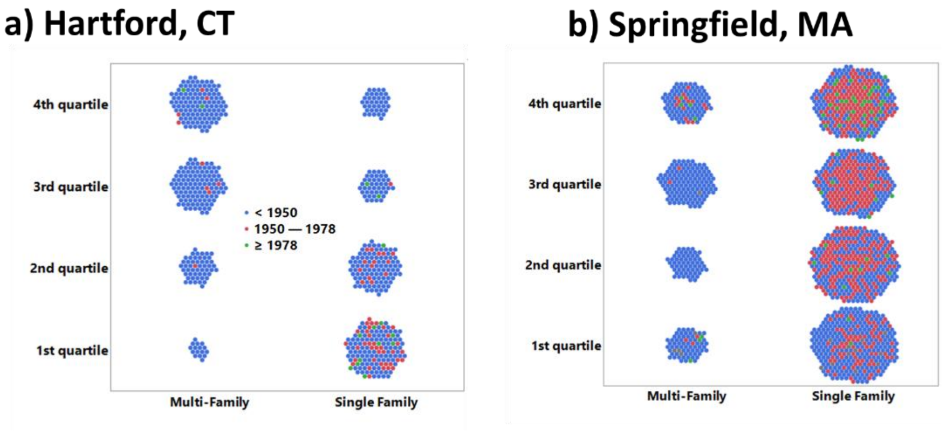

***

Figure S16. Misclassification Rate

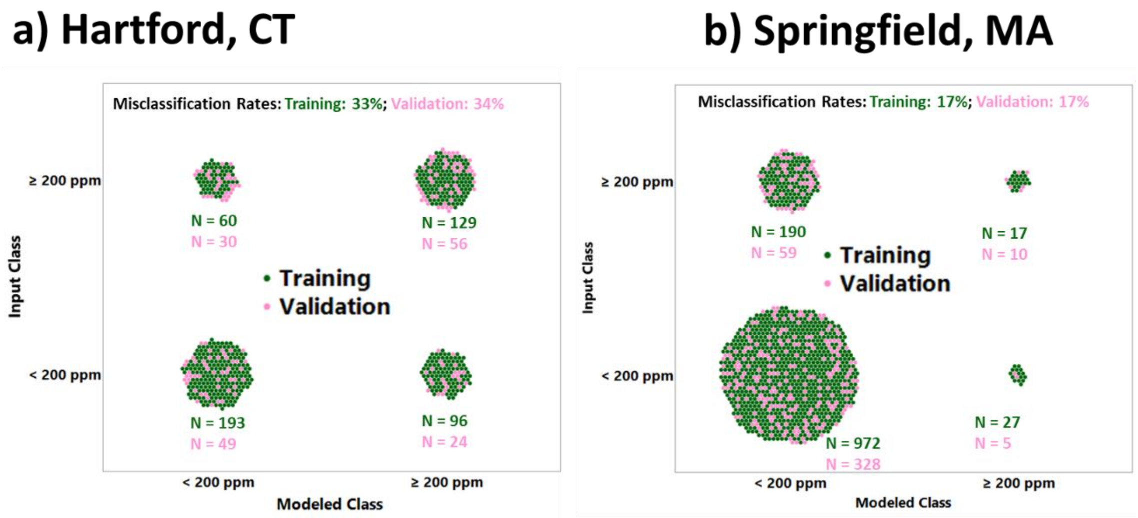

***

Figure S17. Hartford partition model with HLOC rating

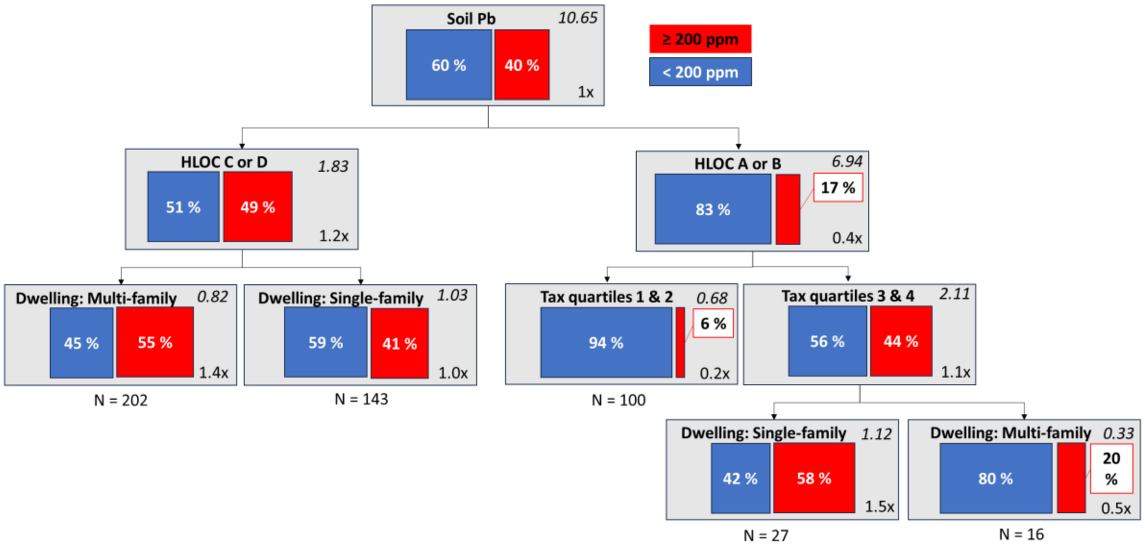

Supplement: Supplementary file 1 — Supporting Information S1 [file GH2-10-e2026GH001854-s001.pdf]
